# Promotion of the inflammatory response in mid colon of complement component 3 knockout mice

**DOI:** 10.1038/s41598-022-05708-8

**Published:** 2022-02-01

**Authors:** Yun Ju Choi, Ji Eun Kim, Su Jin Lee, Jeong Eun Gong, You Jeong Jin, Ho Lee, Dae Youn Hwang

**Affiliations:** 1 Department of Biomaterials Science (BK21 FOUR Program), College of Natural Resources and Life Science/Life and Industry Convergence Research Institute, Pusan National University, Miryang, 50463 Korea; 2 Graduate School of Cancer Science and Policy, Research Institute, National Cancer Center, Goyang, 10408 Korea; 3 Longevity & Wellbeing Research Center/Laboratory Animals Resources Center, Pusan National University, Miryang, 50463 Korea; 4 Department of Biomaterials Science (BK21 FOUR Program), College of Natural Resources and Life Science/Life and Industry Convergence Research Institute/Laboratory Animals Resources Center, Pusan National University, 1268-50 Samrangjin-ro, Samrangjin-eup, Miryang-si, Gyeongsangnam-do 50463 Korea

**Keywords:** Cell biology, Genetics, Immunology, Molecular biology

## Abstract

To determine whether complement component 3 (C3) deficiency affects its receptor downstream-mediated inflammatory response, the current study was undertaken to measure alterations in the inducible nitric oxide synthase (iNOS)‑mediated cyclooxygenase‑2 (COX‑2) induction pathway, inflammasome pathway, nuclear factor-κB (NF-κB) activation, and inflammatory cytokine expressions in the mid colon of C3 knockout (KO) mice. Significant enhancement was observed in expressions of key components of the iNOS‑mediated COX‑2 induction pathway, and in the phosphorylation of mitogen‑activated protein (MAP) kinase members. A similar pattern of increase was also observed in the expression levels of inflammasome proteins in C3 KO mice. Moreover, compared to WT mice, C3 KO mice showed remarkably enhanced phosphorylation of NF-κB and Inhibitor of κB-α (IκB-α), which was reflected in entirety as increased expressions of Tumor necrosis factor (TNF), IL-6 and IL-1α. However, the levels of E-cadherin, tight junction channels and ion channels expressions were lower in the C3 KO mice, although myeloperoxidase (MPO) activity for neutrophils was slightly increased. Taken together, results of the current study indicate that C3 deficiency promotes inflammatory responses in the mid colon of C3 KO mice through activation of the iNOS‑mediated COX‑2 induction pathway, Apoptosis-associated speck-like protein containing a caspase recruitment domain (ASC)-inflammasome pathway and NF-κB signaling pathway, and the enhancement of inflammatory cytokine expressions.

## Introduction

The complement system plays a key role in the opsonization of pathogens and injured cells, induction of inflammation, and destruction of microorganisms in the innate immune system of various organs, including the heart, lung, liver, kidney and gut^1,2^. These responses of the complement are mediated via four routes, viz., the classical, lectin, alternative and thrombin mediated pathways, subsequently resulting in elimination of the antigenic agent and activation of the inflammatory response^2,3^.

During activation of the classical and alternative pathways, C3 is important for regulating various innate immune responses including promotion of opsonic phagocytosis, regulation of humoral immune response, and some T-cell biology^4^. C3 convertase is formed with fragments produced in the classical and lectin pathways (C4bC2b, formerly C4b2a) or the alternative pathway (C3bBb). The C3 convertase then splits the C3 protein into C3a and C3b via proteolytic activity^5^. Subsequently, C3a acts as an anaphylatoxin, while C3b participates to form C5 convertase, following which it contributes to formation of the membrane attack complex (MAC) comprising C5b, C6, C7, C8 and polymeric C9^6^. Based on the key role of C3 within the complement cascade, studies have focused on this protein as a target of complement-directed therapeutic intervention^7^.

The correlation between C3 concentration and inflammatory response has a significant role in several gastrointestinal inflammatory diseases. Patients with inflammatory bowel diseases (IBD), such as ulcerative colitis (UC) and Crohn’s disease (CD), show enhanced catabolism and deposition of C3 as well as circulating C3 conversion products at the site of inflammation^8–10^. Stimulation of C3 and IL-6 production has also been detected in basolateral and apical membranes of Caco-2 cells treated with IL-1β^11^. Expressions of IL-17 and C3 mRNA were reported to remarkably increase in the active lesions of UC and CD, and the IL-17 was determined to stimulate an increase of C3 mRNA expression and C3 secretion in colonic subepithelial myofibroblasts (SEMFs)^12^. Moreover, complement deficiency resulted in significantly suppressed tumor development via activation of the intestinal IL-1β/IL-17A axis, in the azoxymethane and dextran sulfate sodium (DDS)-induced colitis-associated colorectal cancer (CAC) model^13^. Furthermore, activation of the C3 receptors (C3R) on the cell membrane, including C3a and C3b receptors (C3aR and CR1), is linked to regulation of the inflammatory response within various cells. C3aR activation amplifies the MAPK signaling pathway, leading to enhanced expression of proinflammatory cytokines. However, the involvement of iNOS and COX-2 proteins is yet to be examined^14,15^. This activation signal induces NLRP inflammasome activation via increased Extracellular signal-regulated protein kinases 1 and 2 (ERK1/2) and interaction with the NF-κB activation^15,16^. Addition of C3 is reported to stimulate increased expressions of iNOS, IL-6 and IL-1β, and decreases the TGF-β and TNF expressions^17^. However, alterations in the C3R downstream-mediated inflammatory response subsequent to C3 deficiency in the mid colon of mice are poorly understood, although the complement cascade has been considered a novel therapeutic target for IBD.

The current study examines an alternative regulation of the C3R downstream-mediated inflammatory response during C3 deficiency. Our study especially focuses on regulation of the iNOS‑mediated COX‑2 induction pathway, ASC-inflammasome pathway, NF-κB phosphorylation, and inflammatory cytokine expression in the mid colon of C3 KO mice.

## Results

### C3 protein deficiency in kidneys, spleen and thymus of C3 KO mice

To verify the deficiency in major C3 producing organs, expression levels of the C3 protein were measured in the kidney, spleen and thymus of C3 KO mice. Compared to levels obtained in wild type (WT) mice, we obtained decreased expression levels of the C3 protein (Supplementary Fig. 1C), indicating that expression of the C3 protein is successfully inhibited in the kidney, spleen and thymus of C3 KO mice generated using the CRISPR/Cas9-mediated technique.

### Reverse expression pattern of C3 and C3R proteins in the mid colon of C3 KO mice

**Figure 1:**
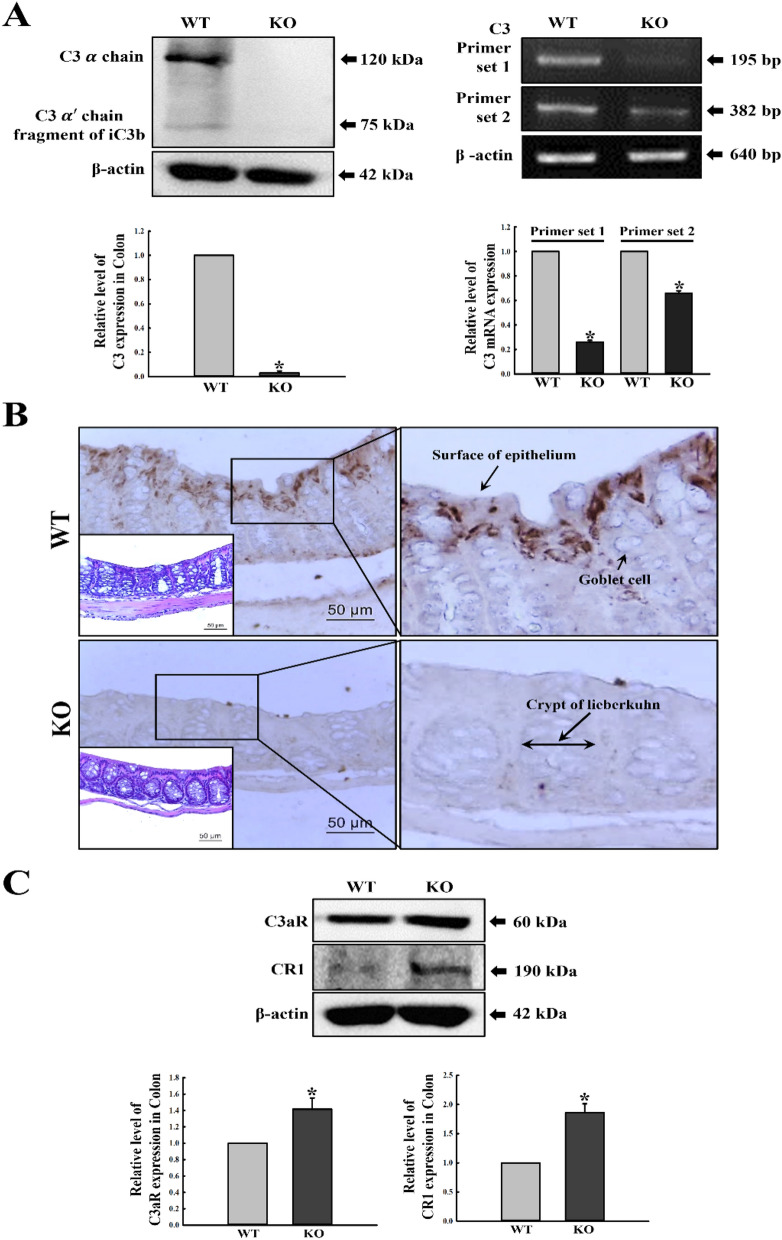
Expression levels of C3 protein and mRNA in the mid colon of C3 KO mice. (**A**) The expressions of C3 protein and mRNA in the mid colon were measured by applying Western blot and RT-PCR analysis, using anti-C3 antibody and C3 specific primers. After determining the intensity of each band using an imaging densitometer, relative levels of the C3 protein were calculated, based on the intensity of β-actin. The mRNA level of the C3 gene was calculated based on the intensity of β-actin as an endogenous control. Tissue samples were collected from 3 to 5 mice per group, and each lysate was analyzed in duplicate for Western blot and RT-PCR analysis (final n = 6–10). (**B**) Tissue distribution of C3 protein was analyzed in the mid colon of WT and C3 KO mice. The C3 protein-specific antibody-stained sections of the mid colon from the WT and KO mice were observed at 400× magnification using light microscopy. The large image in the right column is a magnified image of the rectangle in the left column. H&E-stained sections (low rectangle in left corner) were observed at 400× magnification using a light microscope. (**C**) The expressions of C3aR and CR1 protein in the mid colon were measured with Western blot analysis using anti-C3aR and CR1 antibodies. After determining the intensity of each band using an imaging densitometer, relative levels of C3aR and CR1 proteins were calculated, based on the intensity of β-actin. Tissue samples were collected from 3 to 5 mice per group, and each lysate was analyzed in duplicate for Western blot (final n = 6–10). Data are reported as the mean ± SD. * indicates *p* < 0.05 compared to the WT mice.

To investigate whether C3 deficiency in the mid colon is similar to the levels observed in major C3 producing organs, we measured for expression levels of the C3 protein and mRNA in mid colon samples harvested from C3 KO mice. Compared to levels obtained in WT mice, a similar decrease was observed in the expression levels of the protein and mRNA (Fig. 1A). Moreover, tissue distribution of the C3 protein was detected in the intestinal epithelium of the mid colon of WT mice, whereas no significant color change was observed in sections obtained from C3 KO mice. Considering sensitivity of the antibody used, the color signals observed in the colon tissue section indicate the presence of both full length C3 and C3a fragment (Fig. 1B). However, C3aR and CR1 protein levels revealed a reverse pattern in the Western blot analysis using specific antibodies. C3aR antibody is specific to the 401–482 fragment of the mouse C3aR protein, whereas CR1 antibody produced by immunogen (D109-Q957 region) of human CD35/CR1 recombinant protein recognizes CR1-190, rather than CR1-125 and 150^18^. Both expression levels were higher in the mid colon of C3 KO mice than WT mice (Fig. 1C). Furthermore, the expression levels of C3aR and CR1 proteins were observed to vary by cell type. C3aR proteins were expressed in intestinal epithelial cells (IEC18), nerve cells (B35) and muscle cells (primary cells), and were predominantly high in epithelial cells. Conversely, CR1 proteins were expressed in nerve and epithelial cells, but rarely in muscle cells (Supplement Fig. 4).

### Regulatory effects of C3 deficiency on MAPK signaling pathway

**Figure 2:**
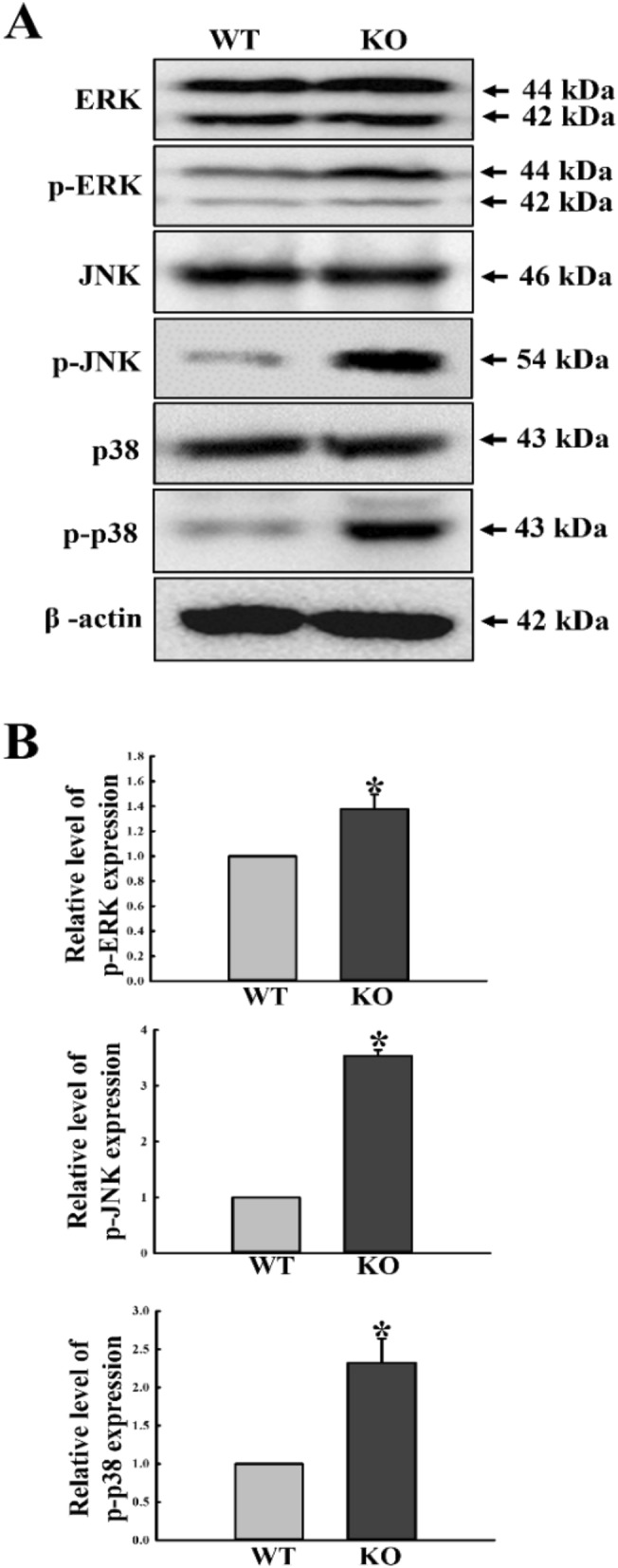
Expression levels of MAPK signaling pathway components. (**A**) Expression levels of ERK, p-ERK, JNK, p-JNK, p38 and p-p38 proteins were determined by Western blot analysis using the specific primary antibody and HRP-labeled anti-rabbit IgG antibody. (**B**) Band intensities were determined using an imaging densitometer, and protein expressions were calculated relative to the intensity of β-actin. Tissue samples were collected from 3 to 5 mice per group, and each lysate was analyzed in duplicate for Western blot (final n = 6–10). Data are reported as the mean ± SD. * indicates *p* < 0.05 compared to the WT mice.

Since the MAPK signaling pathway mediates the transfer of signals derived from C3R^14,15^, we first investigated whether C3 deficiency affects regulation of the MAPK signaling of the C3R downstream pathways in mid colon. To achieve this, alterations in the phosphorylation of ERK, c-Jun N-terminal kinase (JNK) and p38 were measured in the colon of C3 KO mice. As shown in Fig. 2, phosphorylation levels of the three members in the MAPK signaling pathway are remarkably enhanced in KO mice, although the increase rates differed for each protein. The highest protein phosphorylation level was observed for JNK and p38. These results indicate that the MAPK signaling C3R downstream pathways may be activated in the mid colon during C3 deficiency.

**Figure 3:**
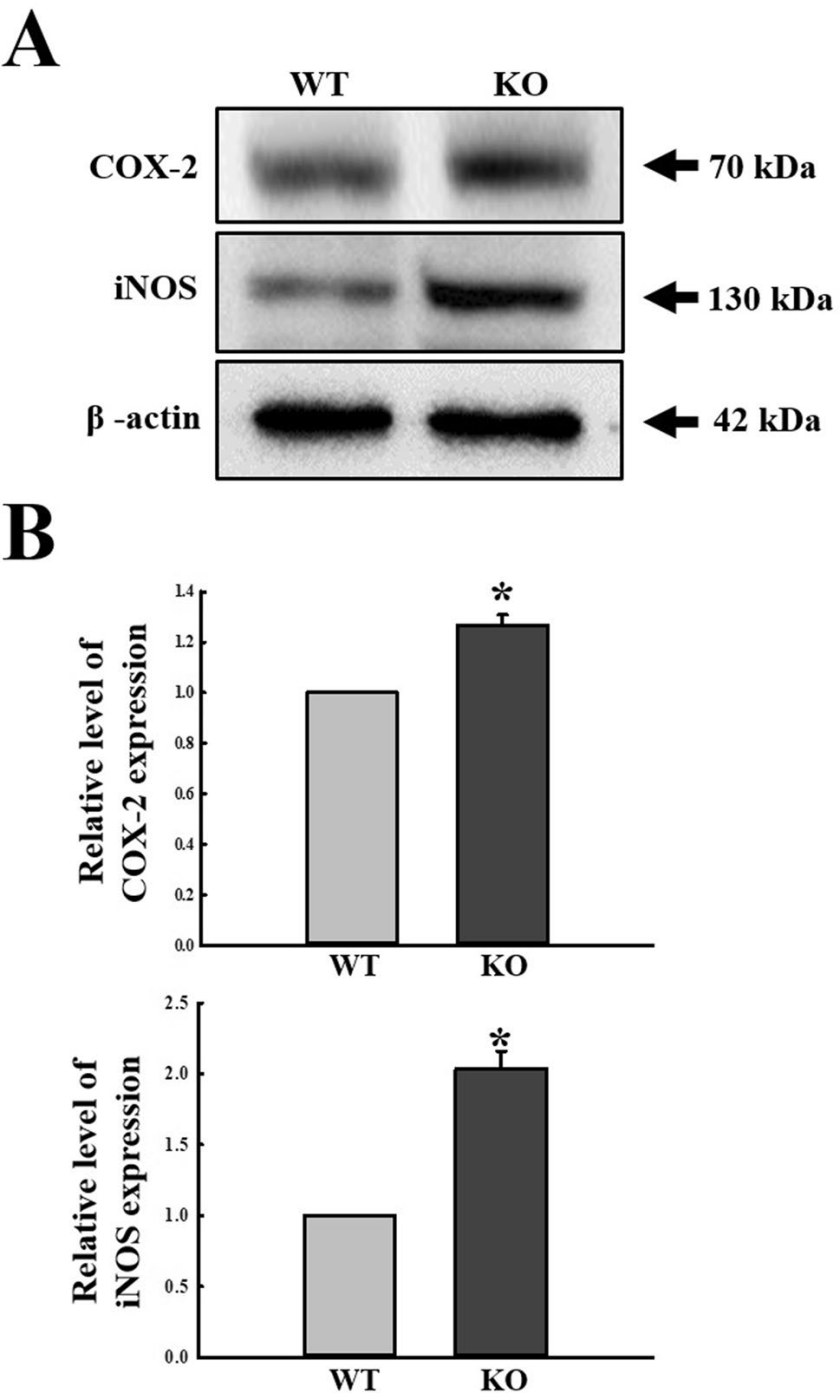
Expression levels of members in the iNOS‑mediated COX‑2 induction pathway. (**A**) Expression levels of COX-2 and iNOS proteins were determined by Western blot analysis using specific primary antibody and HRP-labeled anti-rabbit IgG antibody. (**B**) Band intensities were determined using an imaging densitometer, and protein expressions were calculated relative to the intensity of β-actin. Tissue samples were collected from 3 to 5 mice per group, and each lysate was analyzed in duplicate for Western blot (final n = 6–10). Data are reported as the mean ± SD. * indicates *p* < 0.05 compared to the WT mice.

To investigate whether activation of the MAPK signaling pathway in C3R downstream pathways is accompanied with changes in the iNOS‑mediated COX‑2 induction pathway during C3 deficiency, alterations in the expression levels of iNOS and COX-2 were measured in the mid colon of C3 KO mice. A similar pattern of regulation was observed in both mediators of the iNOS‑mediated COX‑2 induction pathway. The expression levels of both COX-2 and iNOS proteins were significantly increased in the mid colon of C3 KO mice as compared to the WT mice, although the rate of increase was varied (Fig. 3). These results indicate that activation of the MAPK signaling pathway and iNOS‑mediated COX‑2 induction pathway in C3R downstream pathways is tightly linked to C3 deficiency in the mid colon of C3 KO mice.

### Regulatory effects of C3 deficiency on the ASC-inflammasome pathway

**Figure 4:**
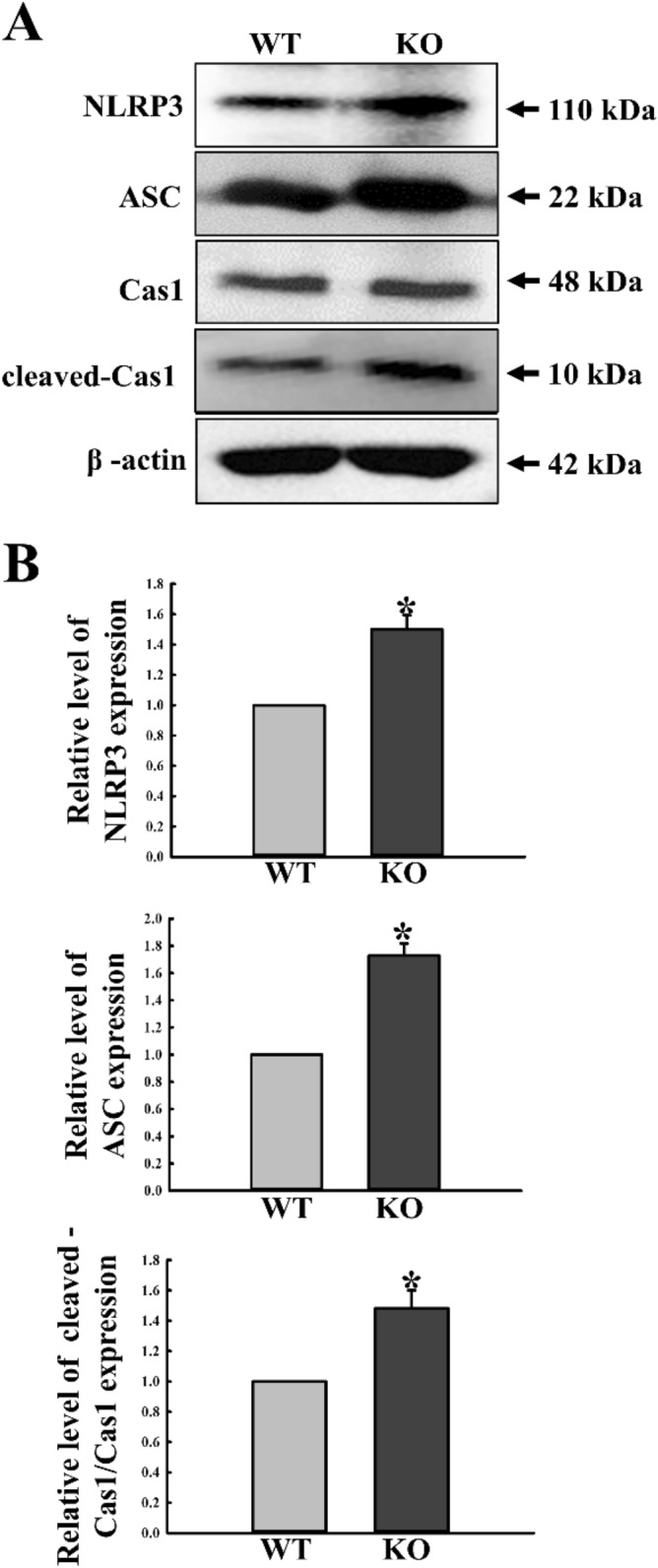
Expression levels of members in the ASC-inflammasome pathway. (**A**) Expression levels of NLRP3, cleaved-Cas1/Cas1 and ASC proteins were determined by Western blot analysis using the specific primary antibody and HRP-labeled anti-rabbit IgG antibody. (**B**) Band intensities were determined using an imaging densitometer, and protein expressions were calculated relative to the intensity of β-actin. Tissue samples were collected from 3 to 5 mice per group, and each lysate was analyzed in duplicate for Western blot (final n = 6–10). Data are reported as the mean ± SD. * indicates *p* < 0.05 compared to the WT mice.

To investigate whether C3 deficiency affects regulation of the ASC-inflammasome pathway of the C3R downstream pathways in mid colon, altered expressions of NLR family pyrin domain containing 3 (NLRP3), cleaved-caspase (Cas1)/Cas1 and ASC were measured in the mid colon of C3 KO mice. ASC expression levels were remarkably increased in the KO mice, as compared to WT mice. A similar pattern was observed for the expressions of the three inflammasomal proteins (Fig. 4). These results indicate that C3 deficiency is associated with upregulation of the ASC-inflammasome pathway of the C3R downstream pathways.

### Regulatory effects of C3 deficiency on the NF-κB signaling pathway

**Figure 5:**
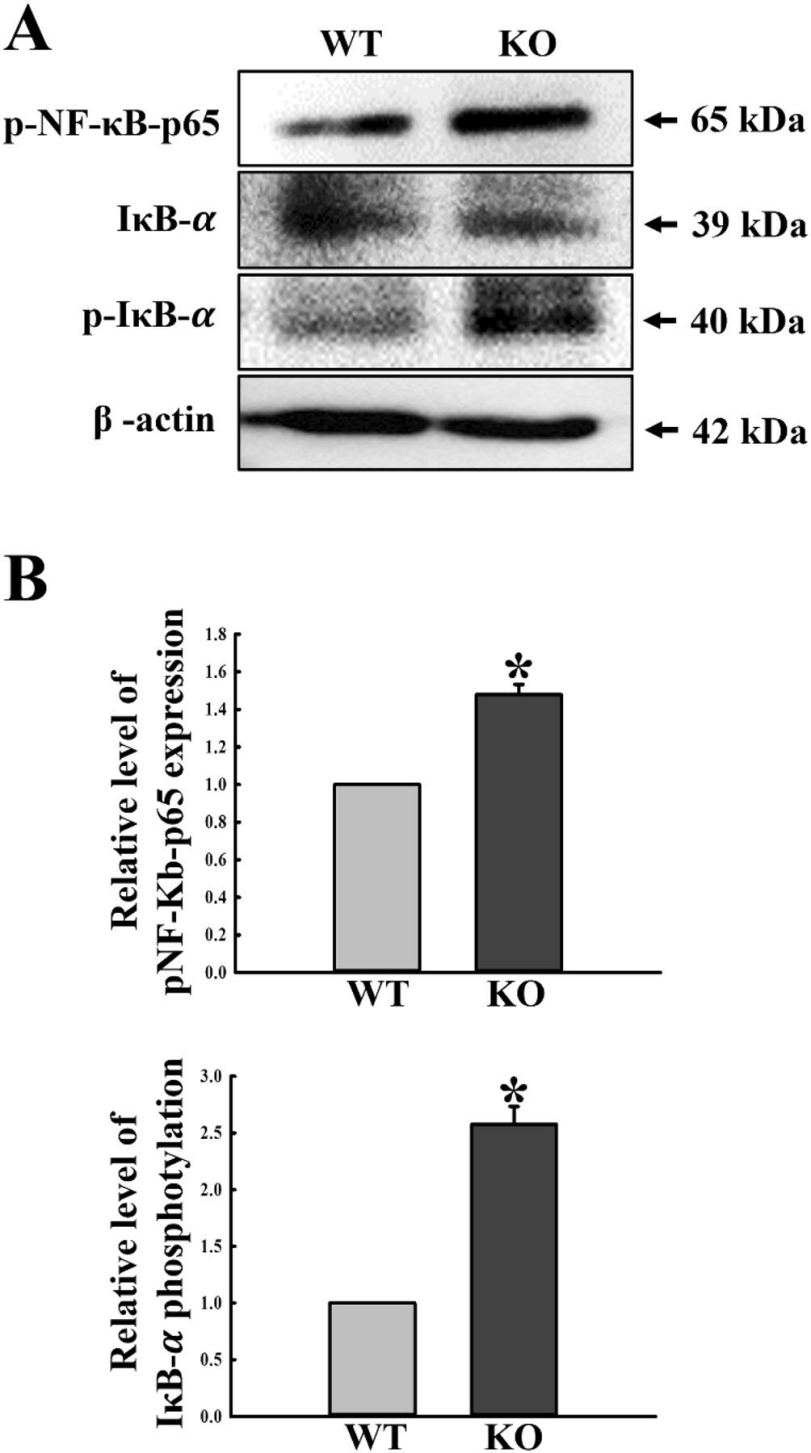
Expression levels of members in the NF-κB signaling pathway. (**A**) Expression levels of NF-κB-p65 and IκB-α proteins were determined by Western blot analysis using specific primary antibody and HRP-labeled anti-rabbit IgG antibody. (**B**) Band intensities were determined using an imaging densitometer, and protein expressions were calculated relative to the intensity of β-actin. Tissue samples were collected from 3 to 5 mice per group, and each lysate was analyzed in duplicate for Western blot (final n = 6–10). Data are reported as the mean ± SD. * indicates *p* < 0.05 compared to the WT mice.

The NF-κB pathway is important for transcriptional regulation of numerous genes involved in the host immune and inflammatory response, and also for the proliferation and survival of cells^19^. We examined whether upregulation of the ASC-inflammasome pathway and activation of iNOS‑mediated COX‑2 induction pathway is accompanied by changes in the NF-κB signaling pathway during C3 deficiency. To achieve this, we measured for altered phosphorylation levels of NF-κB-p65 and IκB-α in the colon of C3 KO mice. As shown in Fig. 5, phosphorylation levels of the two members in the NF-κB signaling pathway were remarkably enhanced in KO mice, although the increase rates differed for both proteins, with highest phosphorylation level obtained for IκB-α. These results indicate that activation of the NF-κB signaling pathway regulated by upregulation of the ASC-inflammasome pathway and iNOS‑mediated COX‑2 induction pathway is associated with C3 deficiency in the mid colon of C3 KO mice.

### Regulatory effects of C3 deficiency on pro-inflammatory and anti-inflammatory cytokines

**Figure 6:**
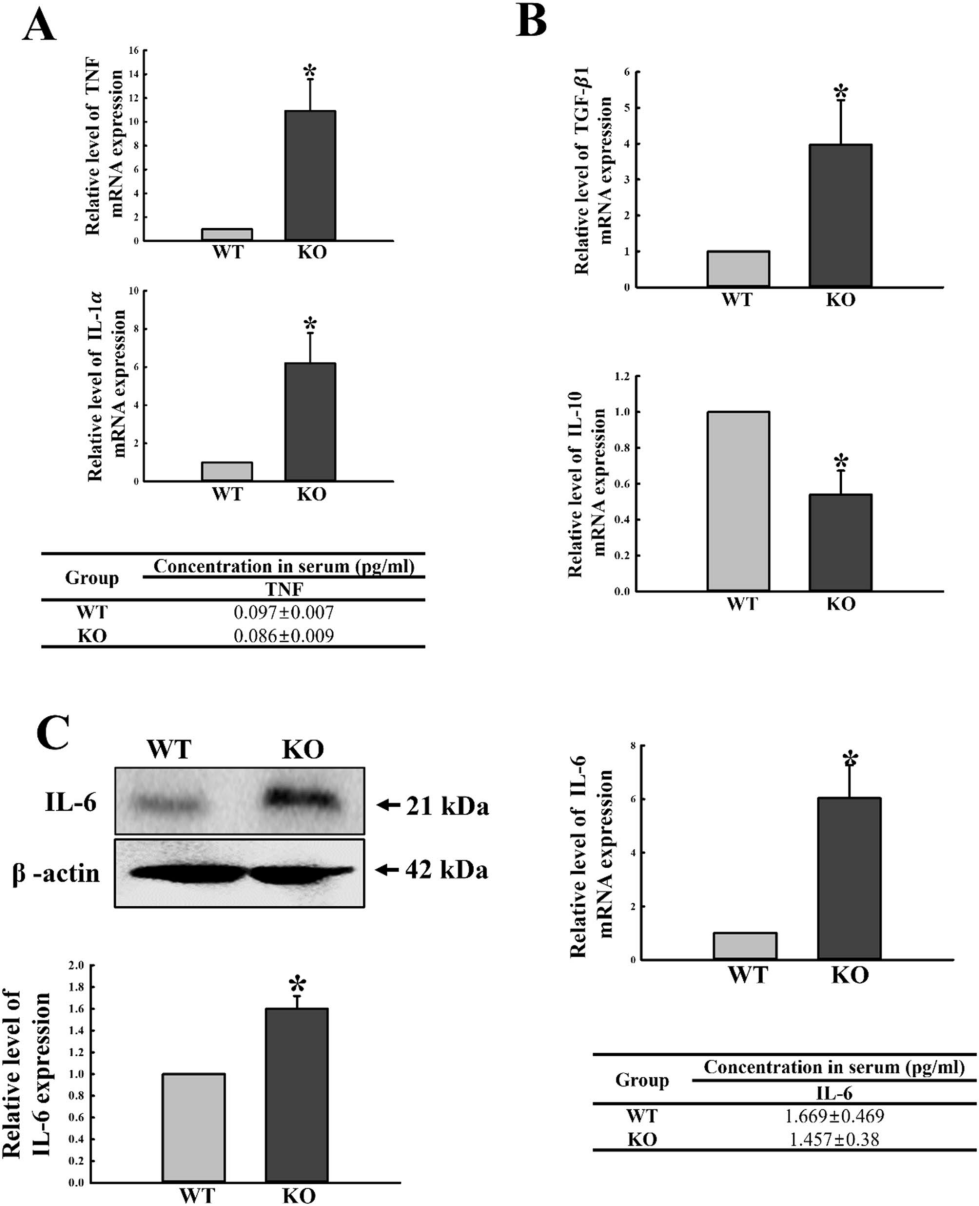
Levels of pro-inflammatory and anti-inflammatory cytokines. (**A**) The levels of TNF and IL-1α transcripts in the total mRNA of colon tissue were measured by qRT-PCR analyses using sense and anti-sense primers set for TNF and IL-1α. Concentration of the TNF protein was measured in the serum of WT and C3 KO mice using the ELISA Kit. This assay detects concentrations as low as 3.5 pg/mL for TNF. (**B**) The levels of anti-inflammatory cytokines including TGF-$\beta$ 1 and IL-10 transcripts in the total mRNA of colon tissue were measured by qRT-PCR analyses using the sense and anti-sense primer set for TGF-β and IL-10. The mRNA level of each gene was calculated based on the intensity of actin as an endogenous control. Tissue samples were collected from 3 to 5 mice per group, and each lysate was analyzed in duplicate for qRT-PCR (final n = 6–10). (**C**) Expression levels of IL-6 protein were determined by Western blot analysis using specific primary antibody and HRP-labeled anti-rabbit IgG antibody. Band intensities were determined using an imaging densitometer, and protein expressions were calculated relative to the intensity of actin. Tissue samples were collected from 3 to 5 mice per group, and each lysate was analyzed in duplicate for ELISA (final n = 6–10). The concentrations of IL-6 proteins were measured in the serum of WT and C3 KO mice using the ELISA Kit. This assay detects concentrations as low as 2 pg/mL for IL-6. Data are reported as the mean ± SD. * indicates *p* < 0.05 compared to the WT mice.

We further examined whether activation of the NF-κB signaling pathway during C3 deficiency leads to regulation of pro-inflammatory and anti-inflammatory cytokine expressions in the mid colon. To achieve this, the transcript levels of TNF, IL-1α, TGF-β1, IL-10 and IL-6 were evaluated by quantitative real-time (qRT)-PCR of mid colon samples obtained from C3 KO mice. A similar pattern of regulation was observed for the two pro-inflammatory cytokines (TNF and IL-1α). The mRNA levels of both cytokines were remarkably increased in the mid colon of C3 KO mice, as compared to WT mice. However, the protein levels of TNF were constantly maintained in the serum of both WT and C3 KO mice (Fig. 6A). Meanwhile, a different pattern was detected in the expression of anti-inflammatory cytokines. The mRNA levels of TGF-β1 were increased, whereas the levels of IL-10 were decreased (Fig. 6B). Moreover, the protein and mRNA expressions of IL-6, a pro-inflammatory cytokine as well as an anti-inflammatory cytokine, were significantly increased in the mid colon of C3 KO mice. However, the concentration of IL-6 in serum remained constant, although the low levels measured were below the sensitivity of the assay (2 pg/mL) (Fig. 6C). These results indicate that activation of the NF-κB signaling pathway during C3 deficiency probably mediates upregulation of the inflammatory cytokines in the mid colon of C3 KO mice.

### Role of compensatory adaptive pathway for C5 convertase to increase inflammatory mediators during C3 deficiency

**Figure 7:**
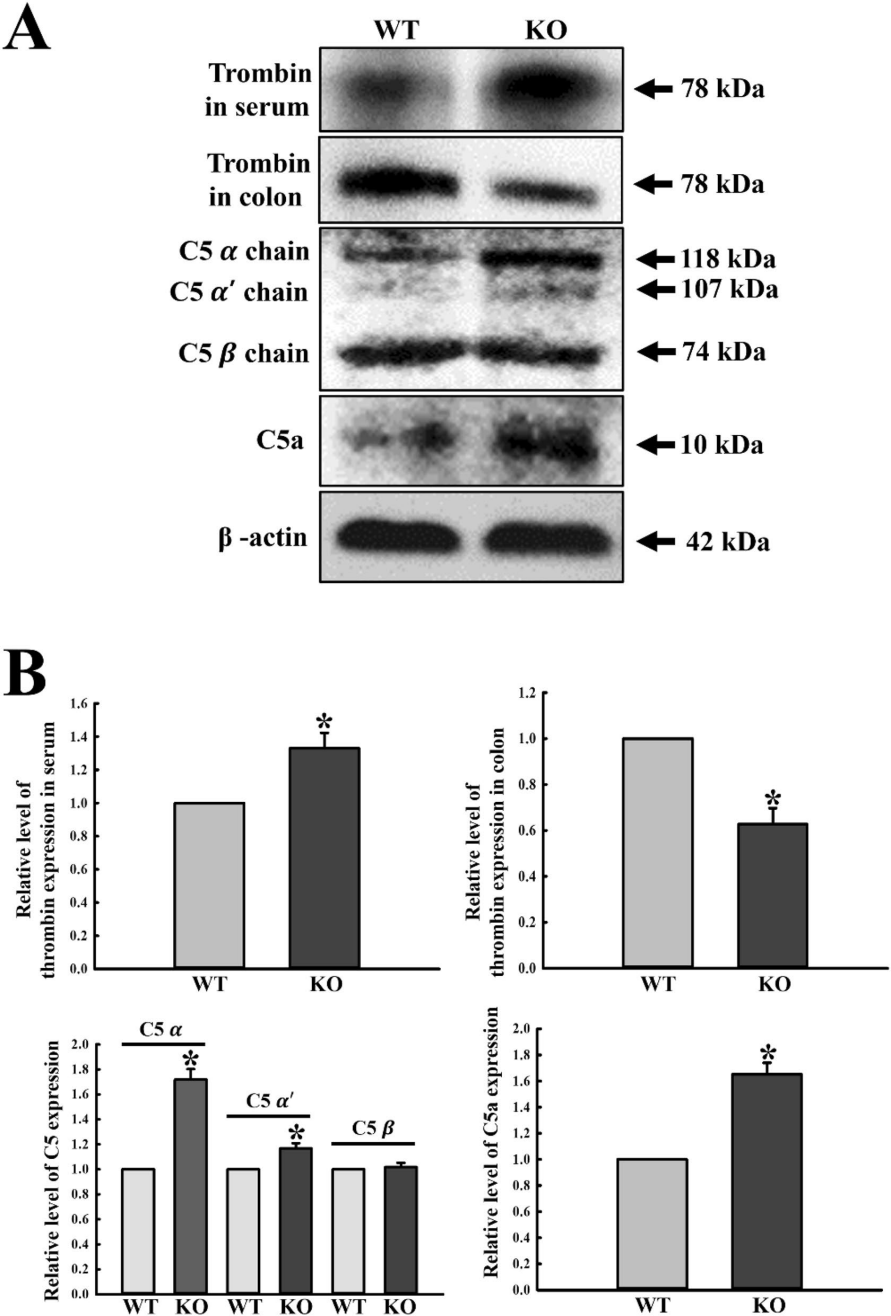
Levels of C5 and its mediated inflammatory regulators. (**A**) Expression levels of thrombin and C5 proteins were determined by Western blot analysis using specific primary antibody and HRP-labeled anti-rabbit IgG antibody. (**B**) Band intensities were determined using an imaging densitometer, and protein expressions were calculated relative to the intensity of β-actin. Tissue samples were collected from 3 to 5 mice per group, and each lysate was analyzed in duplicate for Western blot (final n = 6–10). Data are reported as the mean ± SD. * indicates *p* < 0.05 compared to the WT mice.

The consistent production of C5a in C3 KO mice is mediated by thrombin, which functions as a potential C5 convertase^3^. We, therefore, investigated whether increased inflammatory mediators in C3 KO mice are associated with the consistent production of C5a through a compensatory adaptive pathway for C5 convertase. To achieve this, alterations in the expression levels of thrombin and C5 were measured in the serum and mid colon of C3 KO mice. Compared to WT mice, the expression levels of thrombin proteins were significantly decreased in the colon tissue of C3 KO mice, while levels were enhanced in the serum obtained from same mice (Fig. 7). Moreover, the expression levels of C5a protein were higher in the C3 KO mice than WT mice (Fig. 7). These results indicate that during C3 deficiency, increased inflammatory mediators are associated with maintaining the concentration of C5 cleaved products through overproduction of thrombin, which acts as C5 convertase.

### Effects of C3 deficiency on infiltration of neutrophils and leaky epithelium of the mid colon

**Figure 8:**
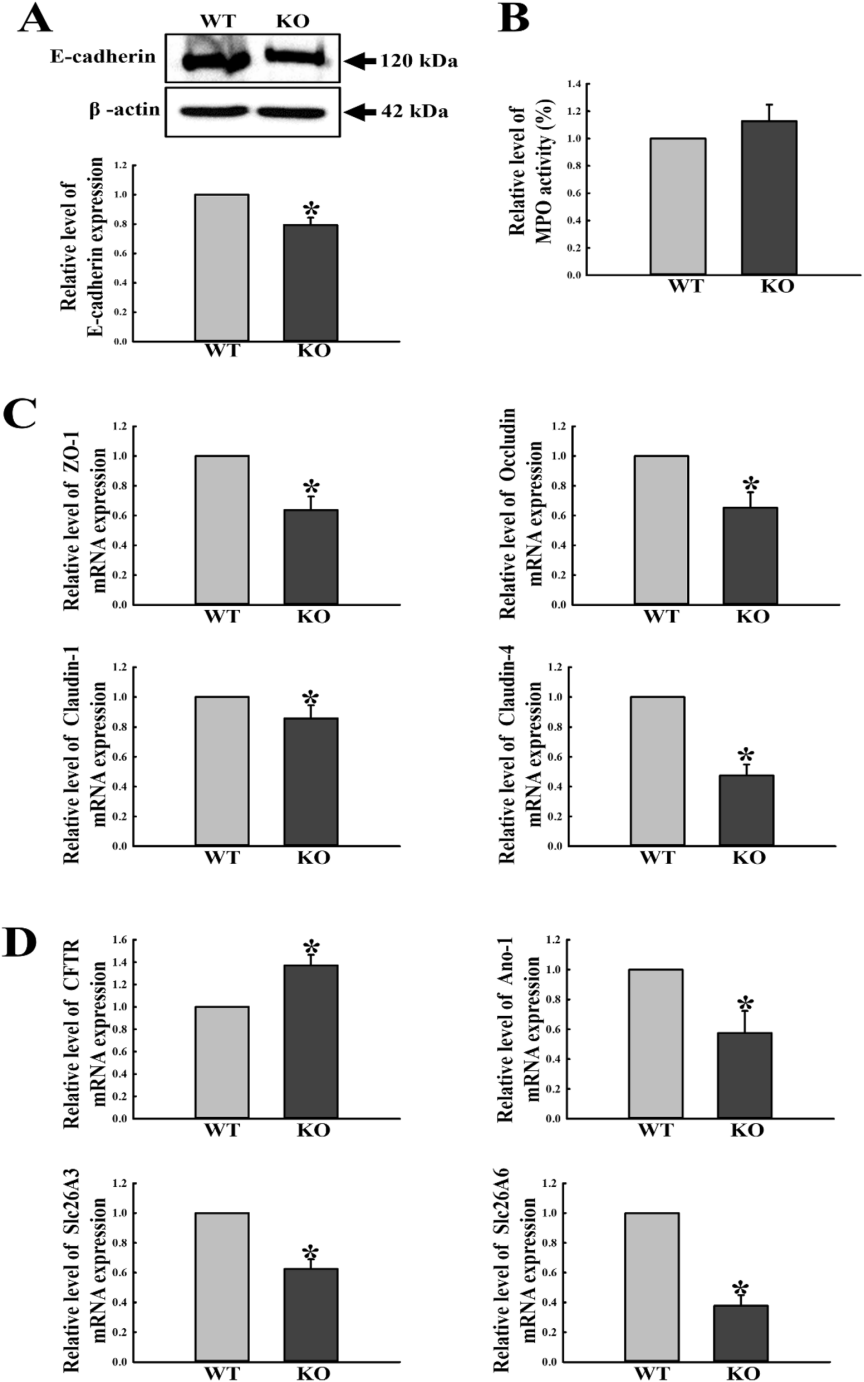
Alterations in neutrophil infiltration and leaky epithelium. (**A**) Expression levels of E-cadherin were determined by Western blot analysis using specific primary antibody and HRP-labeled anti-rabbit IgG antibody. Band intensities were determined using an imaging densitometer, and protein expressions were calculated relative to the intensity of β-actin. Tissue samples were collected from 3 to 5 mice per group, and each lysate was analyzed in duplicate for Western blot (final n = 6–10). (**B**) MPO activity for neutrophil level. MPO activity was measured in lysate of mid colon tissues using the MPO assay kit. Tissue samples were collected from 3 to 5 mice per group, and each lysate was analyzed in duplicate for ELISA (final n = 6–10). (**C**) The levels of four tight junction channels including ZO-1, Occludin, Claudin-1 and Claudin-4 transcripts in the total mRNA of colon tissue were measured by qRT-PCR analyses using sense and anti-sense primers set for ZO-1, Occludin, Claudin-1 and Claudin-4. (**D**) The levels of four tight junction channels including CFTR, Ano-1, Slc26A3 and Slc26A6 transcripts in the total mRNA of colon tissue were measured by qRT-PCR analyses using sense and anti-sense primers set for CFTR, Ano-1, Slc26A3 and Slc26A6. The mRNA level of each gene was calculated based on the intensity of actin as an endogenous control. Tissue samples were collected from 3 to 5 mice per group, and each lysate was analyzed in duplicate for qRT-PCR (final n = 6–10). Data are reported as the mean ± SD. * indicates *p* < 0.05 compared to the WT mice.

To investigate whether C3 deficiency-induced inflammation is accompanied with other features of inflammation including neutrophil infiltration and leaky epithelium, we evaluated alterations in the levels of cell adhesion proteins, tight junction channels, ion channels and neutrophils by Western blot, qRT-PCR and ELISA, in the mid colon of C3 KO mice. The expression levels of E-cadherin and four tight junction channels (ZO-1, Occludin, Claudine-1 and Claudine-4) were remarkably decreased in the mid colon of C3 KO mice as compared to WT mice (Fig. 8A,C). A similar pattern was detected for the expression levels of three ion channels, including Ano-1, Slc26A3 and Slc26A6. However, the mRNA level of one other ion channel (CFTR) was enhanced in the mid colon of C3 KO mice, as compared to WT mice (Fig. 8D). Furthermore, the MPO activity indicating the neutrophil level was constantly maintained in both the C3 KO and WT mice, although it was slightly higher in the KO than WT mice (Fig. 8B). Taken together, the above results indicate that C3 deficiency-induced inflammation is associated with dysregulation on gut homeostasis, resulting in leaky epithelium of the mid colon in C3 KO mice.

## Discussion

Chronic gastrointestinal inflammation is the most common inflammatory response linked to the development of various IBDs, including CD and UC^20^. Complications of long-term inflammation are proposed to be a major contributor to the development of colorectal cancer (CRC). Diverse physiological events occurring in the gastrointestinal mucosa and lumen during the inflammatory response include cell activation, cytokine production, complement activation and tissue damage^21,22^. The regulatory factors assessed in this study are considered important for maintaining the balance between pro- and anti-inflammatory responses, which is the driving force behind inflammation and immune response to treat these diseases^20^. We therefore undertook to investigate the effects of C3 deficiency on the C3R downstream-mediated inflammatory response of the gastrointestinal tract, by analyzing alterations in the iNOS‑mediated COX‑2 induction pathway, ASC-inflammasome pathway, NF-κB signaling pathway, and inflammatory cytokine expression, in the mid colon of C3 KO mice. Results of the present study provide first evidence that C3 deficiency may be tightly linked with upregulation of the inflammatory response of the C3R downstream signaling pathway, in mid colon of C3 KO mice. This study provides further evidence that the absence of C3 induces various immune problems in the gut, although deficiencies in key proteins (C1–C4) in the early process of the complement pathway are known to cause autoimmune diseases, including systemic lupus erythematosus^23^. Furthermore, these results indicate that activation of C3 can be considered an important factor during IBD. However, more studies are required to verify the molecular mechanism of C3 and C3 receptors in the mid colon of C3 KO mice.

Several split products of the complement, including C3a and C5a, play a major role in regulating the immune system activity including degranulation, extravasation and chemotaxis, as well as activation of the immune cells and non-myeloid cells^24,25^. Considering all factors of the complement, C3a is known to have conflicting functions in the inflammatory response. Briefly, C3a contributes to the pro-inflammatory response, including enhancement of the G-protein coupled C3aR expression in immune cells and non-myeloid cells, induction of oxidative burst in macrophages, and stimulation of histamine release from basophils and mast cells^25–28^. However, the anti-inflammatory role was also investigated in ischemia–reperfusion injury and in the sepsis model showing acute phase of inflammation^29,30^. Especially, C3a prevents the migration and degranulation of neutrophils, although other granulocytes are activated^31^. Furthermore, significant alterations were observed in the expression levels of inflammatory cytokines in C3 KO mice. The cytokine ratio between levels of IL-10 and interferon (IFN)-γ or IL-17 shows a shift in the jejunum of ovalbumin challenged C3 KO mice^32^. Increased TNF and IL-12 levels were greater in the colon of DSS-treated C3 KO mice than DSS-treated WT mice^33^. In the current study, the iNOS‑mediated COX‑2 induction pathway and inflammatory cytokine expressions were upregulated in the mid colon of C3 KO mice. These results provide additional evidence that C3 deficiency plays an alternative role in the inflammatory response of mid colon, although further studies are required to determine the molecular mechanism.

In the current study, we measured the mRNA level of some cytokines in the mid colon of C3 KO mice. Enhanced levels of TNF, IL-6 and IL-1α transcripts were observed in the mid colon during C3 deficiency, although the rate of increase was widely varied. It is impossible to directly compare our findings with previous results since the disease model used in each study was different. However, few studies have revealed a correlation between C3 and inflammation in the gastrointestinal tract. Previous studies also reported significant alterations in the levels of cytokines and C3 protein in IBD. C3 metabolism (including cleavage, circulation and deposition) was significantly increased in IBD, while levels of IL-17 and C3 mRNA were enhanced in UC and CD^8–10,12^. Furthermore, IL-1β-induced C3 and IL-6 production was observed at the apical or basolateral membrane or chamber of CaCo-2 cells^11^. A similar response was also observed in macrophages after treatment with C3 peptides, where increased iNOS, IL-6 and IL-1β expressions and decreased TGF-β and TNF expressions were determined^17^.

Oxidative stress is a condition in which the balance of oxidative stimulators and inhibitors in the body is disturbed by events such as inflammation. This ultimately causes oxidative damage to cells and the human body^34^. During this balancing, the iNOS-mediated COX‑2 induction pathway is considered a key regulatory mechanism, since expressions of iNOS and COX-2 proteins are induced by numerous pro‑inflammatory stimuli (such as LPS and TNF) in various diseases^35,36^. The overexpression and activation of iNOS promote the production of nitric oxide (NO), which stimulates the activation of COX‑2^37^. This process, mediated by the NF-κB and MAPK signaling pathways, has a critical role in the regulation of cell growth and differentiation, as well as in the control of cellular responses to cytokines and stresses^38–40^. Also, the above inflammatory pathway regulated by the iNOS-mediated COX‑2 induction pathway is observed to improve with diet components (including fibers, polyphenols, and poly-unsaturated fatty acids) as well as lifestyle changes (including fasting and physical exercise)^41^. In the current study, we measured whether the iNOS-mediated COX-2 induction pathway of the C3R downstream pathway can be activated by C3 deficiency in the mid colon. Compared to the WT mice, we observed significantly enhanced expression levels of COX-2 and iNOS in the mid colon of C3 KO mice. These results provide additional evidence that the role of C3 in the mid colon is associated with the iNOS-mediated COX-2 induction pathway, although we were unable to directly compare our findings to previous studies.

Furthermore, the inflammatory response in gut is said to be associated with the difference in the composition of microbiota. Numerous immune-mediated inflammatory diseases are reported to exhibit a significant change in the composition of the gut microbiota^42^. The difference of gut microbiota has also been linked to the pathogenesis of chronic inflammatory diseases^43–45^. Moreover, our recent study revealed remarkable changes in the proportions of several microbial genera detected in fecal microbiota of 16-week-old C3 KO mice with constipation phenotypes^46^. Therefore, the impact on the composition of microbiota needs to be considered when researching the inflammatory mechanisms of C3 in the mid colon of mice. However, in the current study, we used 7-week-old C3 KO and WT mice bred in SPF conditions to exclude the effects of gut microbes in the two strains. Nevertheless, since the experimental design did not cohouse the two strains to share a similar microbial population, this can be considered a limitation of our research. Furthermore, the control of animal microbiome by cohousing may be considered as one of the reasons for upregulation of several signaling molecules, since the cells with the elevated levels were not verified in our study.

**Figure 9:**
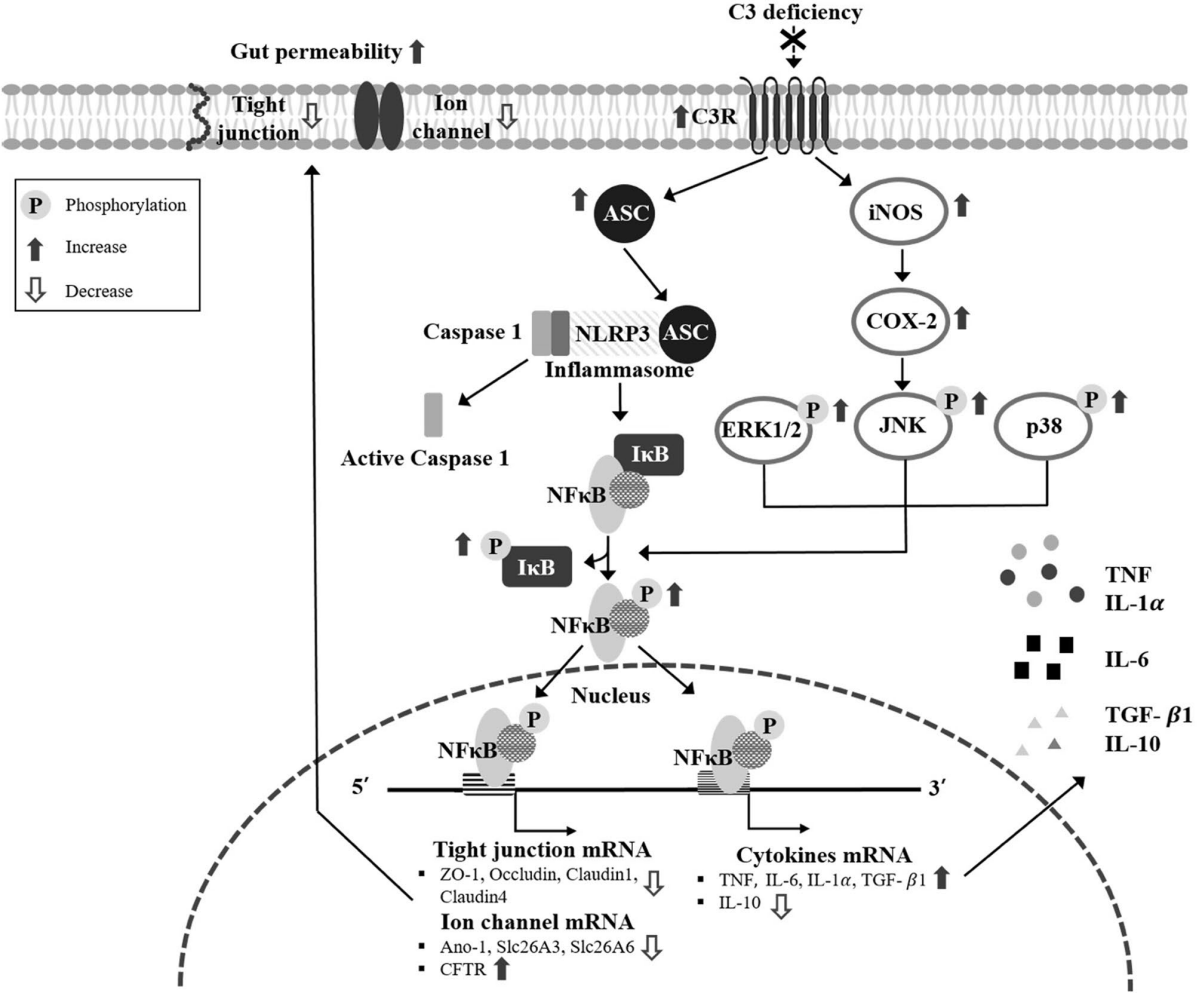
Suggested mechanism of inflammatory response in the C3R downstream pathway during C3 deficiency. In this scheme, the deficiency of C3 is thought to activate the iNOS-mediated COX-2 induction pathway, MAPK signaling pathway, ASC-inflammasome pathway, and NF-κB signaling pathway. Finally, activated NF-κB translocates into the nucleus, and induces the upregulation of inflammatory cytokines as well as the downregulation of tight junction channels and ion channels in the mid colon.

Taken together, our study undertook to investigate the effects of C3 deficiency on the C3R downstream signaling pathway-mediated inflammatory response in the mid colon. Our results indicate that C3 deficiency induces the upregulation of inflammatory cytokines through activation of the iNOS-mediated COX-2 induction pathway, ASC-inflammasome pathway, and NF-κB signaling pathway, in the mid colon of C3 KO mice (Fig. 9). Furthermore, we provide additional evidence for the role of C3 in inflammatory responses and mucosal damage to the colon. However, this study provides limited information on the correlation between C3 deficiency and C3R downstream-mediated inflammatory response since the functional validation for C3 deficiency was not analyzed in the mid colon of C3 KO mice, which therefore needs to be clarified by gathering more experimental evidence.

## Materials and methods

### Animal care and use

All experiments and methods were performed in accordance with relevant guidelines and regulations. The experiments complied with the ARRIVE guidelines. Protocol for the C3 KO mice study was approved by the Pusan National University-Institutional Animal Care and Use Committee (PNU-IACUC; Approval Number PNU-2020-2657). The C3 KO and WT mice were handled at the Pusan National University-Laboratory Animal Resources Center, which is accredited by the Korea Food and Drug Administration (FDA) (Accredited Unit Number: 000231), and Association for Assessment and Accreditation of Laboratory Animal Care (AAALAC) International (Accredited Unit Number: 001525). Seven-week-old C3 KO (n = 7) and WT (n = 7) mice having the Friend Virus B Type NIH (FVB) genetic background were kindly provided by the Department of Laboratory Animal Resources at the National Institute of Food and Drug Safety Evaluation (NIFDS, Chungju, Korea). The C3 KO mice having an 11-nt deletion mutation in exon 2 of the C3 gene was achieved using the Clustered Regularly Interspaced Short Palindromic Repeats (CRISPR) design tool (crispor.tefor.net) (Supplement Fig. 1A)^47^. Throughout the experimental period, all animals were provided ad libitum access to water, and a standard irradiated chow diet (Samtako BioKorea Co., Osan, Korea) consisting of moisture (12.5%), crude protein (25.43%), crude fat (6.06%), crude fiber (3.9%), crude ash (5.31%), calcium (1.14%), and phosphorus (0.99%). All animals were maintained in a specific pathogen free (SPF) state at 23 ± 2 °C and 50 ± 10% relative humidity under a strict light cycle (lights on at 08:00 h and off at 20:00 h). After adaptation for 1 week, the mice were euthanized using a chamber filled with CO_2_ gas, and mid colons were subsequently harvested for further analysis. The colon region containing soft stools was collected as the mid colon from the total large intestine; watery stools in the proximal colon are sequentially transformed into hard stools in the distal colon via the passage of soft stools in the mid colon^48,49^.

WT and C3 KO mice were identified using tail genomic DNA PCR analysis. After preparation of the reaction mixture, two sets of primers were added to detect the C3 gene: set 1, forward primer (5′-CAR CTG CTC CAG TGA GAA C-3′) and reverse primer (5′-CTT CTC AGA TGT CCA CTG GCT C-3′); set 2, forward primer (5′-CAT CTG CTC CAG CAC YGA GAA C-3′) and reverse primer (5′-TGC CTC TTT AGG AAG TCT TG-3′). All reactions were performed at 38 cycles of amplification on a Perkin-Elmer Thermal Cycler, using the following conditions: 30 s, 94 °C; 30 s, 60 °C; 1 min, 72 °C. After amplification, the 291 bp and 280 bp final PCR products obtained were electrophoresed on 1% agarose gels (Supplement Fig. 1B).

### qRT-PCR

qRT-PCR was applied to assess the relative quantities of inflammatory cytokine mRNAs. Briefly, total RNA molecules were isolated from frozen mid colon tissues using RNA Bee solution (Tet-Test Inc., Friendswood, TX, USA). After quantification of RNA, complement DNA (cDNA) was synthesized using a mixture of oligo-dT primer (Thermo Fisher Scientific Inc., Waltham, MA, USA), dNTP and reverse transcriptase (Superscript II, 18064-014, Thermo Fisher Scientific Inc.). qRT-PCR was then achieved using a cDNA template and 2× Power SYBR Green (TOYOBO Co., Osaka, Japan), as described in a previous study^50^. The primer sequences used to evaluate the mRNA levels were as follows: C3, sense primer 5′-CATAT GCTCC AGCAC TGAGA AC-3′, antisense primer 5′-TGCCT CTTTA GGAAG TCTTG-3′; NF-κB, sense primer 5′-GTAAC AGCAG GACCC AAGGA-3′, antisense primer 5′-AGCCC CTAAT ACACG CCTCT-3′; TNF, sense primer 5′-CCTGT AGCCC ACGTC GTAGC-3′, antisense primer 5′-TTGAC CTCAG CGCTG ACTTG-3′; IL-6, sense primer 5′-TTGGG ACTGA TGTTG TTGAC A-3′, antisense primer 5′-TCATC GCTGT TGATA CAATC AGA-3′; IL-1α, sense primer 5′-CAGTT CTGCC ATTGA CCAT-3′, antisense primer 5′-TCTCA CTGAA ACTCA GCCGT-3′; TGF-$\beta$1, sense primer 5′-GAGGT CACCC GCGTG CTA-3′, antisense primer 5′-TGTGT GAGAT GTCTT TGGTT TTCTC-3′; IL-10, sense primer 5′-CAGCC GGGAA GACAA TAACT G-3′, antisense primer 5′-CCGCA GCTCT AGGAG CATGT-3′; ZO-1, sense primer 5′-CCTCC GTTGC CCTCA CAGTA-3′, antisense primer 5′-GGGCG CCCTT GGAAT G-3′; Occludin, sense primer 5′-TTGAA GAGTG GGTTA AAAAT GTGTC T-3′, antisense primer 5′-TCAAC TCTTT CCGCA TAGTC AGAT-3′; Claudin-1, sense primer 5′-CCCCG GAAAA CAACC TCTTA C-3′, antisense primer 5′-TGTCA CACAT AGTCT TTCCC ACTAG AA-3′; Claudin-4, sense primer 5′-CGTGG CAAGC ATGCT GATTA-3′, antisense primer 5′-GTCGC GGATG ACGTT GTG-3′; CFTR, sense primer 5′-TCTGC CGCGC AGCAA-3′, antisense primer 5′-GGTGT GAACG TCATC AGATC CA-3′; Ano-1, sense primer 5′-GCACG GCTTC GTCAA TCAC-3′, antisense primer 5′-TTGGG TGCTG TGCCA TTCT-3′; Slc26A3, sense primer 5′-TGTTT AATTT GGATC ATGAC CTTCA-3′, antisense primer 5′-TTGCC GCCAG GCCTA A-3′; Slc26A6, sense primer 5′-CTGCT CCAGC ACGTT CCA-3′, antisense primer 5′-CAGAT CACCC AGGAG CCATT-3′; β-actin, sense and antisense primers 5′-TGGAA TCCTG TGGCA TCCAT GAAAC-3′ and 5′-TAAAA CGCAG CTCAG TAACA GTCCG-3′, respectively. The reaction cycle at which PCR products exceeded the fluorescence intensity threshold during the exponential phase of PCR amplification was considered as the threshold cycle (CT).

### Histopathological analysis

Mid colons harvested from mice of the subset groups were fixed in 10% formalin for 48 h, embedded in paraffin wax, and then sectioned into 4 μm thick slices. The colon sections were collected on glass slides and stained with Hematoxylin & Eosin (H&E) (Sigma-Aldrich Co., St. Louis, MO, USA), after which they were examined by light microscopy for histopathology, at 400× magnification.

### Immunohistochemical (IHC) staining analysis

Tissue distribution of the C3 protein was detected by IHC staining using light microscopy, as previously described^51^. Briefly, the mid colon tissue samples were fixed in 10% formalin for 12 h, embedded in paraffin, and sliced into 4 µm thick sections. These sections were subsequently deparaffinized with xylene, rehydrated, and pretreated for 30 min at room temperature with 1× PBS blocking buffer containing 10% goat serum (Vector Laboratories, Burlingame, CA, USA). The sections were then incubated with primary anti-C3 antibody (Abcam Com., Cambridge, UK), diluted 1:300 in 1× PBS blocking buffer. Also, a group treated with only HRP-conjugated secondary antibody without primary antibody was analyzed as control to rule out false signals during the immunohistochemical analysis. The antigen–antibody complexes were visualized with biotinylated secondary antibody (goat anti rabbit)-conjugated horseradish peroxidase (HRP) streptavidin (Histostain-Plus Kit, Zymed, South San Francisco, CA, USA), at a dilution of 1:300 in PBS blocking buffer. Finally, C3 proteins were detected using stable diaminobenzidine (DAB) (Invitrogen Co., Carlsbad, CA, USA) and the Leica Application Suite (Leica Microsystems, Wetzlar, Germany).

### Western blot

Total proteins were extracted from the mid colon of C3 KO and WT mice using the Pro-Prep Protein Extraction Solution (Intron Biotechnology Inc., Seongnam, Korea). After centrifugation of tissue homogenates at 13,000 rpm for 5 min, protein concentrations were determined using a Pierce BCA Protein Assay Kit (Thermo Fisher Scientific Inc.). Proteins (30 µg) were then separated by 4–20% sodium dodecyl sulfate–polyacrylamide gel electrophoresis (SDS-PAGE) for 2 h, and subsequently transferred to nitrocellulose membranes for 3 h at 40 V. Each membrane was then incubated separately, overnight at 4 °C, with the following primary antibodies: anti-C3 (ab200999, Abcam Com.), anti-C3aR (bs-2955R, Bioss Inc.), anti-CR1 (LS-C777464, LSBio Inc.), anti-COX-2 (12282, Cell Signaling Technology Inc., Danvers, MA, USA), anti-NLRP3 (15101, Cell Signaling Technology Inc.), anti-Cas 1 (24232, Cell Signaling Technology Inc.), anti-ASC (67824, Cell Signaling Technology Inc.), anti-iNOS (PA3-0304, Thermo Fisher Scientific Inc.), anti-ERK1/2 (9102, Cell Signaling Technology Inc.), anti-p-ERK (E-4) (9101, Santa Cruz Biotechnology Inc., Dallas, TX, USA), anti-JNK (9252, Cell Signaling Technology Inc.), anti-p-JNK (9251, Cell Signaling Technology Inc.), anti-p38 (9212, Cell Signaling Technology Inc.), anti-p-p38 (9211, Cell Signaling Technology Inc.), anti-p-NF-κB-p65 (A00284T254, Boster Bio Inc., Pleasanton, CA, USA), anti-IκB (9242, Cell Signaling Technology Inc.), anti-p-IκB (9246S, Cell Signaling Technology Inc.), anti-IL-6 (SC-1265, Santa Cruz Biotechnology Inc.), anti-C5 (ab11898, Abcam Com.), anti-thrombin (ab92621, Abcam Com.), anti-E-cadherin (24E10, Cell Signaling Technology Inc.) or anti-β-actin (4967, Sigma-Aldrich Co.). Membranes were subsequently washed with washing buffer (137 mM NaCl, 2.7 mM KCl, 10 mM Na_2_HPO_4_, 2 mM KH_2_PO_4_, and 0.05% Tween 20) and incubated with 1:2,000 diluted horseradish peroxidase-conjugated goat anti-rabbit IgG (Zymed Laboratories, South San Francisco, CA, USA) at room temperature for 1 h. Blots were developed using a Chemiluminescence Reagent Plus kit (Pfizer Inc., Gladstone, NJ, USA). Chemiluminescence signal band image for each protein was detected using a digital camera (1.92 MP resolution) of the FluorChem FC2 Imaging system (Alpha Innotech Corporation, San Leandro, CA, USA), followed by semi-quantification of protein densities using the AlphaView Program version 3.2.2 (Cell Biosciences Inc., Santa Clara, CA, USA).

### Enzyme-linked immunosorbent assay (ELISA) for evaluating TNF and IL-6 cytokines

Serum concentrations of TNF and IL-6 cytokines were measured using the mouse TNF enzyme-linked immunosorbent assay kit (Biolegend, San Diego, CA, USA) and IL-6 ELISA kit (Biolegend), according to the manufacturer’s protocols. Briefly, serum was isolated from whole blood of each mouse, diluted 1:75,000, and pipetted into designated wells of the kit. The plate was incubated for 20 min at room temperature, followed by washing and addition of 1× enzyme-antibody conjugate. After incubation for 20 min at room temperature and additional washing, tetramethylbenzidine (TMB) substrate was added to each well, and the color alteration was determined using a Vmax plate reader (Molecular Devices, Sunnyvale, CA, USA) at 450 nm.

### Analysis of MPO activity

The activity of MPO in the colon was measured using the MPO assay kit (Elabscience, Wuhan, China). Briefly, a 50 mg piece from the mid colon was homogenized in 1× PBS. The homogenate was mixed with o-dianisidine hydrochloride, 0.0005% hydrogen peroxide and 0.1 mM H_2_O, followed by incubation at 37 °C. After centrifugation at 2325 g for 10 min, supernatants from each sample were indirectly assessed by measuring the OD value at 460 nm. MPO activities are expressed as U/mg protein.

### Statistical analysis

Statistical significance was evaluated using the Student’s unpaired t-test. Data are presented as mean ± standard deviation (SD). *p* < 0.05 is considered to indicate a statistically significant difference.

## Supplementary Information

Supplementary Information.
