# Supplementary material for: Promotion of the inflammatory response in mid colon of complement component 3 knockout mice
Source: Sci Rep. 2022 Feb 1;12:1700. doi: 10.1038/s41598-022-05708-8 (PMC8807838; doi:10.1038/s41598-022-05708-8)
Supplement: Supplementary file 1 — Supplementary Information. [file 41598_2022_5708_MOESM1_ESM.pdf]

Promotion of the inflammatory response in mid colon of complement component 3 knockout mice

Yun Ju Choi[1], Ji Eun Kim[1], Su Jin Lee[1], Jeong Eun Gong[1], You Jeong Jin[1], Ho Lee[2] and Dae Youn Hwang[1,3,*]

[1]*Department of Biomaterials Science (BK21 FOUR Program), College of Natural Resources and Life Science/Life and Industry Convergence Research Institute, Pusan National University, Miryang 50463, Korea*

[2]*Graduate School of Cancer Science and Policy, Research Institute, National Cancer Center, Goyang 10408, Korea*

[3]*Longevity & Wellbeing Research Center/Laboratory Animals Resources Center, Pusan National University, Miryang 50463, Korea*

*Corresponding author: Dae Youn Hwang, Professor and Director, dyhwang@pusan.ac.kr

Supplement Fig. 1

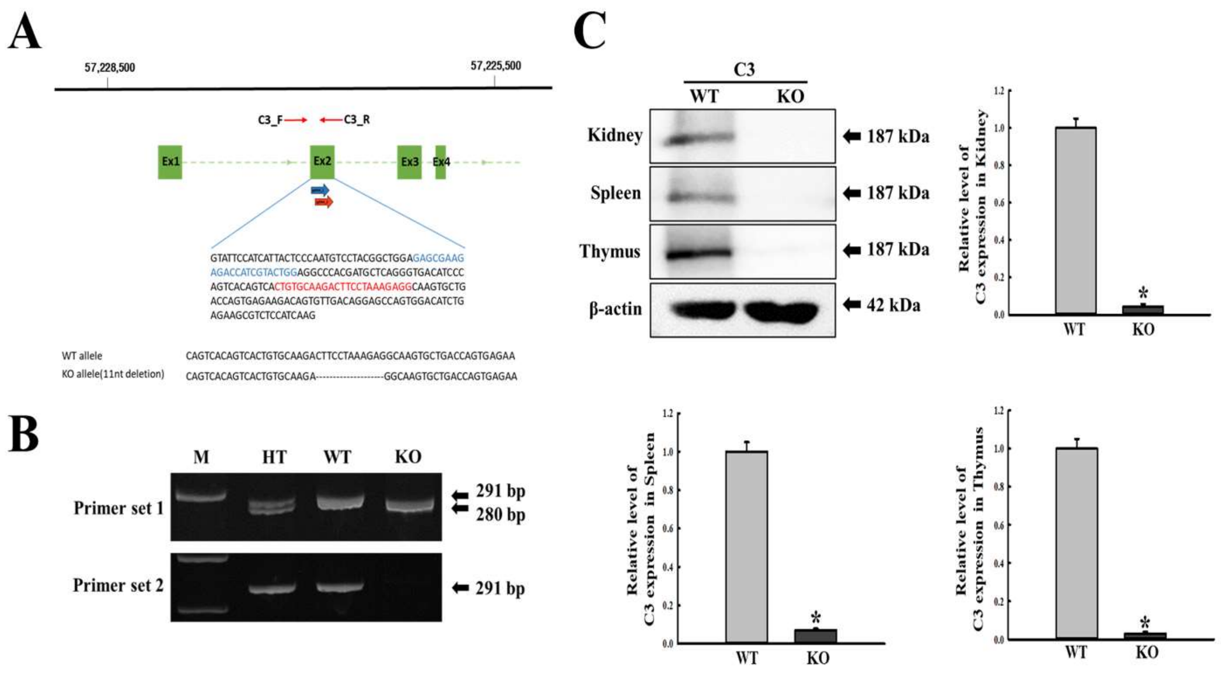

Targeting scheme for the C3 gene, identification of C3 KO mice, and expression of C3 protein. **(A)** 11 nucleotides in exon 2 of the C3 gene was deleted with a mixture of Cas9 protein and 2 sgRNA, as presented in Materials and Methods. **(B)** Deletion of C3 gene was identified by DNA analysis using genomic DNA isolated from tails of founder mice. M and HT indicate the maker and heterogenous type. **(C)** The expressions of C3 protein in the kidney, spleen and thymus tissues were measured with Western blot analysis using anti-C3 antibody and HRP-labeled anti-rabbit IgG antibody. Band intensities were determined using an imaging densitometer, and expressions of the proteins were calculated relative to the intensity of β-actin. Three to five mice per group were used for the preparation of tissue lysates, and Western blots were assayed in duplicate for each sample. Data are reported as the mean ± SD. * indicates $p < 0.05$ compared to the WT mice.

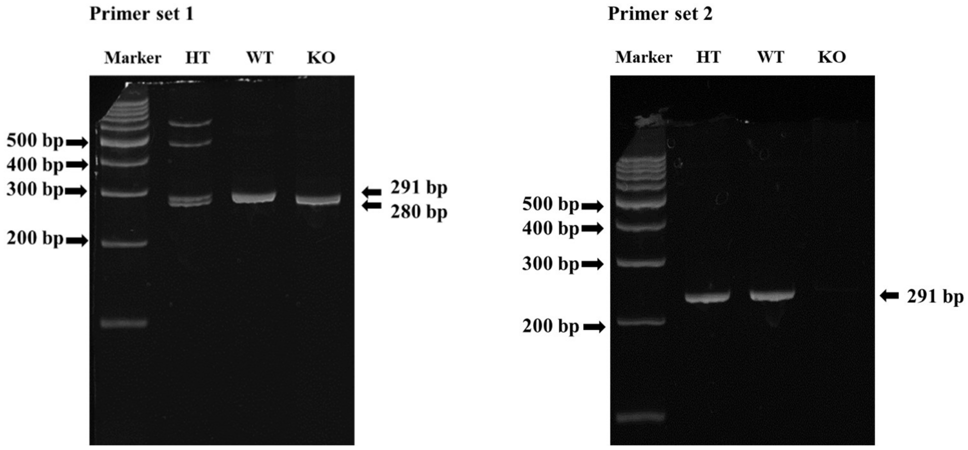

Original images of the gels in Figure S1B.

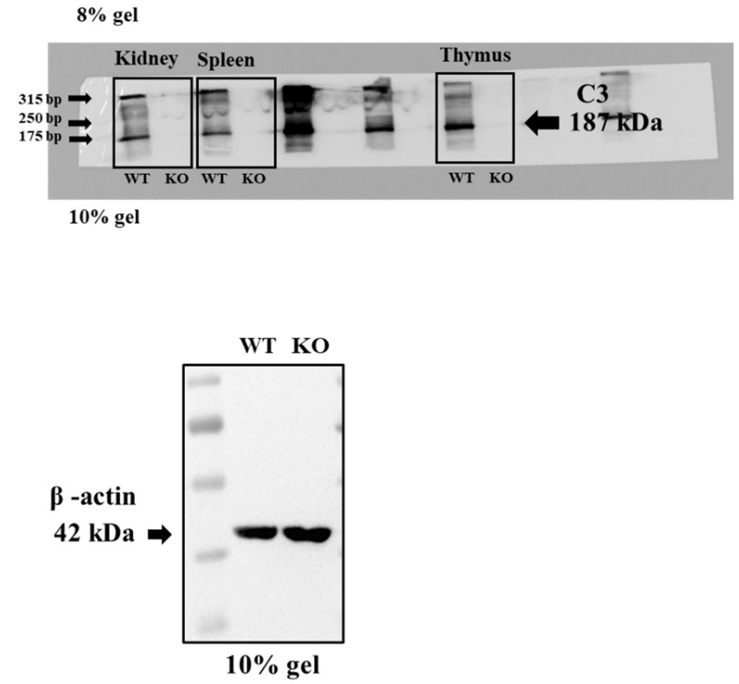

Original images of the gels in Figure S1C.

Supplement Fig. 2

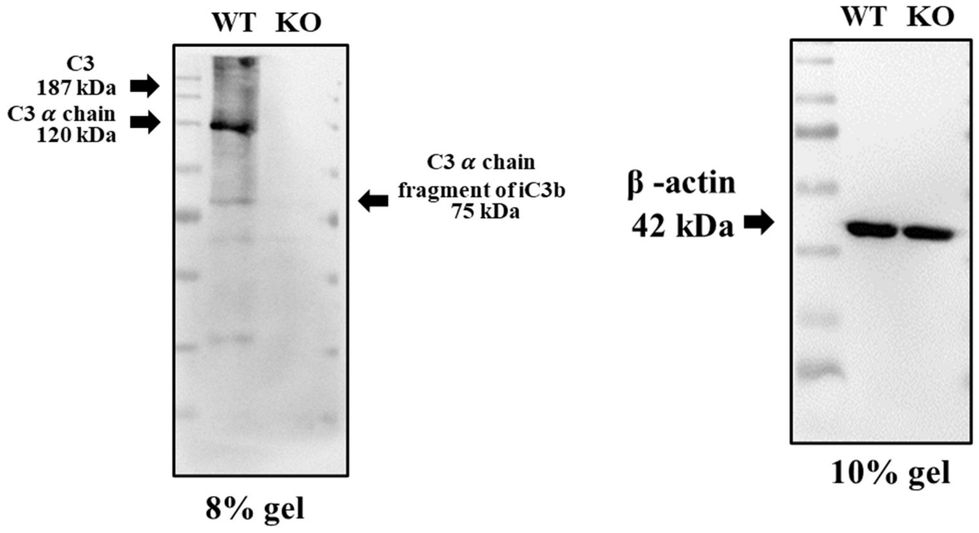

Original image of C3 western blot band.

Supplement Fig. 3

A

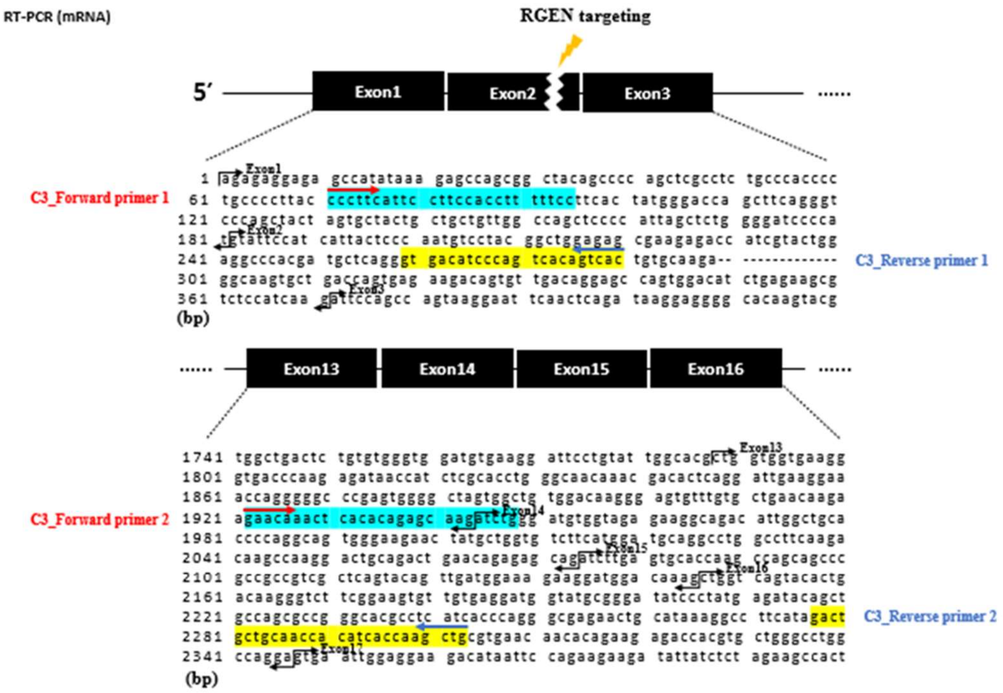

B

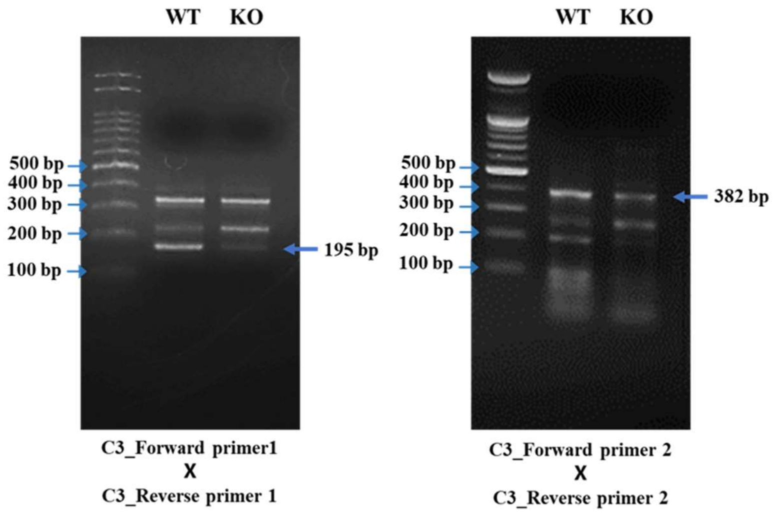

Expression Levels of C3 mRNA in the mid colon of C3 KO mice. (A) Primer sequence of C3 primer set1 and 2. (B) Original image of C3 RT-PCR band.

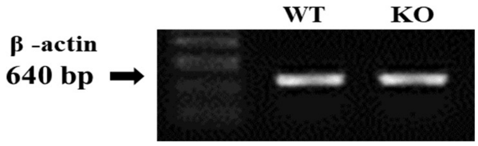

Original images of the gels in Figure 1A.

Supplement Fig. 4

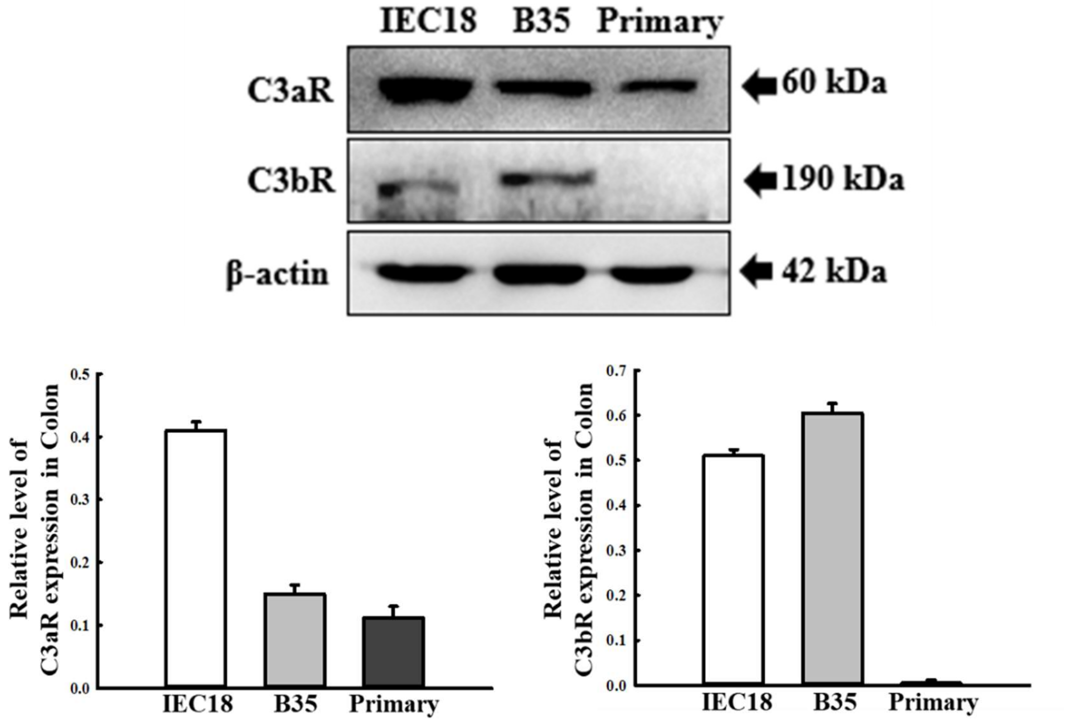

Expression Levels of C3aR and C3bR in IEC18, B35, Primary cells (muscle cell derived from FVB mice). **(A)** Expression levels of C3aR and C3bR proteins were determined by Western blot analysis using specific primary antibody and HRP-labeled anti-rabbit IgG antibody. Band intensities were determined using an imaging densitometer, and expressions of the proteins were calculated relative to the intensity of β-actin. Three to five mice per group were used for the preparation of tissue lysates, and Western blots were assayed in duplicate for each sample. Data are reported as the mean ± SD.

Supplement Fig. 5

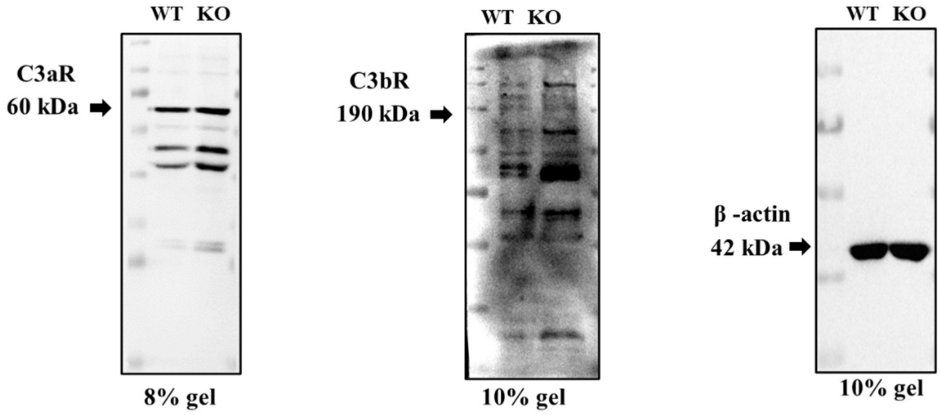

Original images of the gels in Figure 1B.

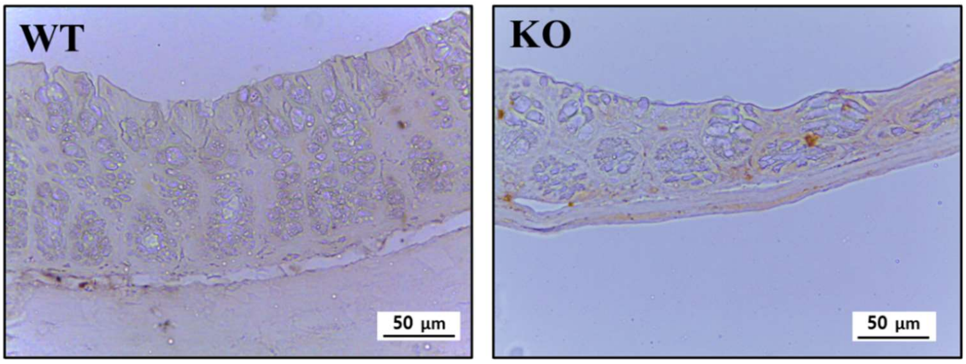

Images of IHC staining using secondary antibody only control in Figure 1C.

Supplement Fig. 6

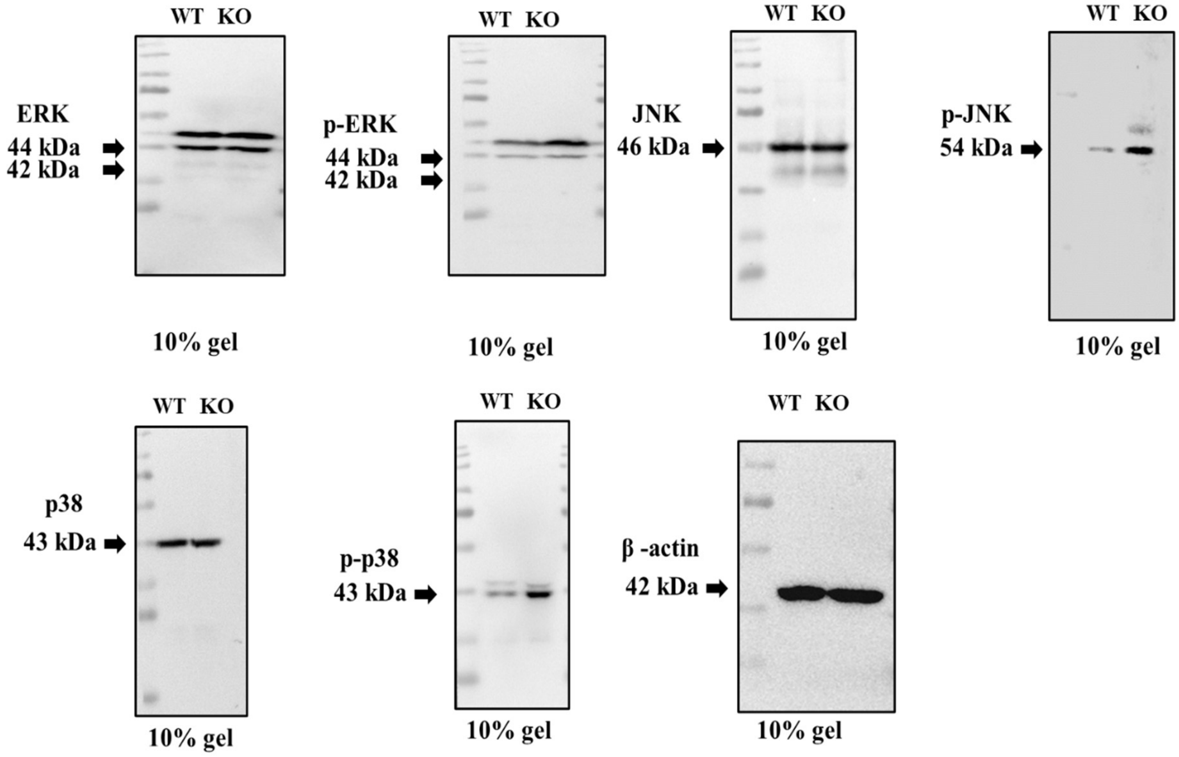

Original images of the gels in Figure 2A.

Supplement Fig. 7

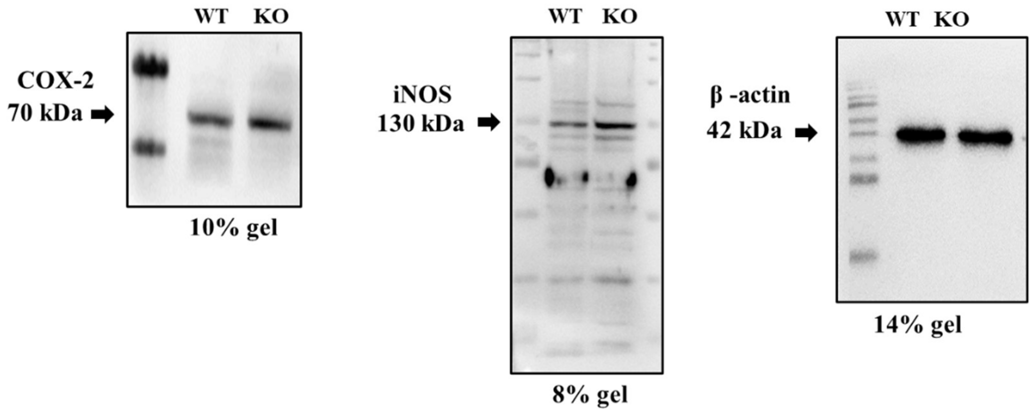

Original images of the gels in Figure 3A.

Supplement Fig. 8

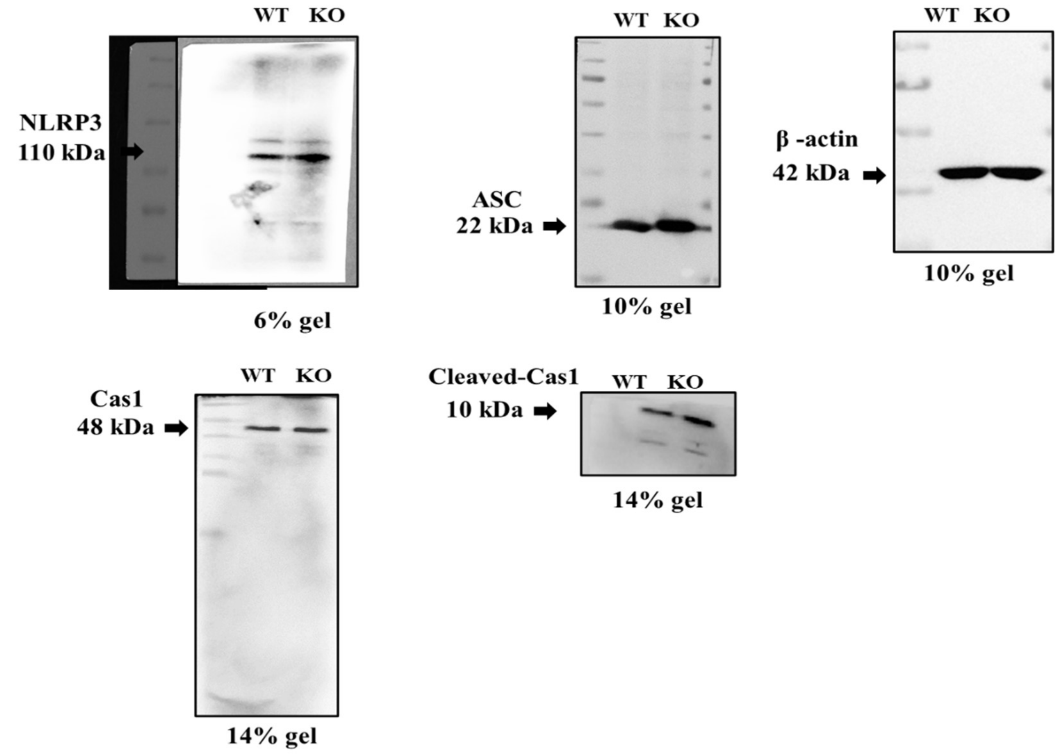

Original images of the gels in Figure 4A.

Supplement Fig. 9

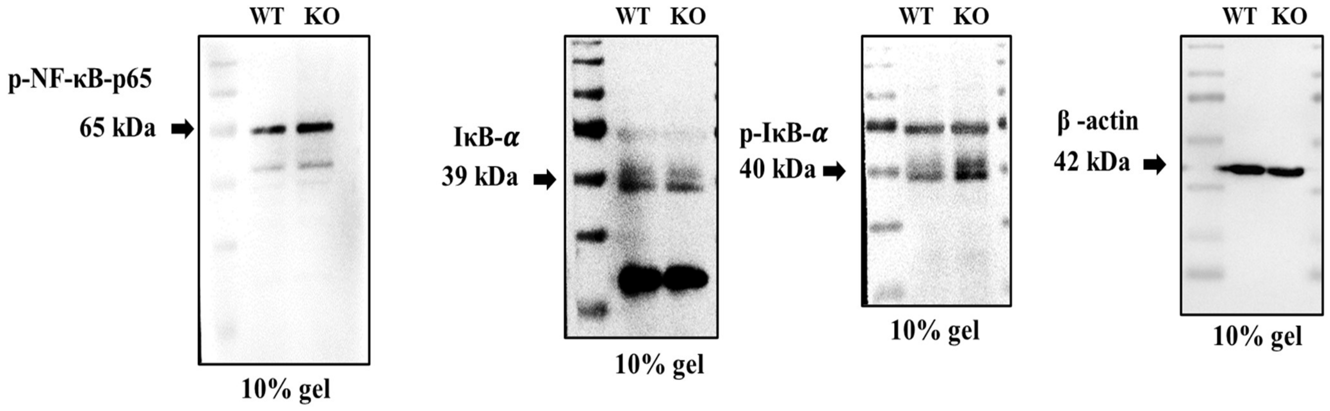

Original images of the gels in Figure 5A.

Supplement Fig. 10

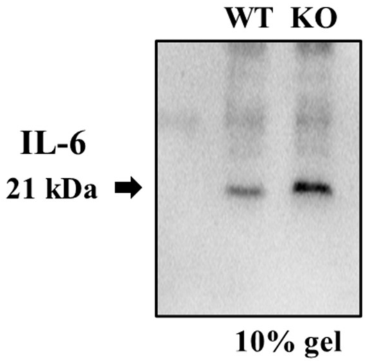

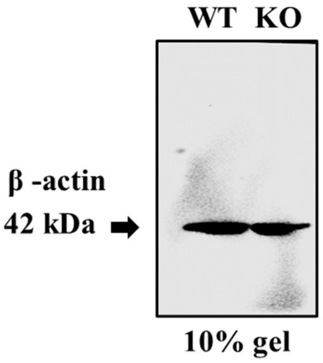

Original images of the gels in Figure 6B.

Supplement Fig. 11

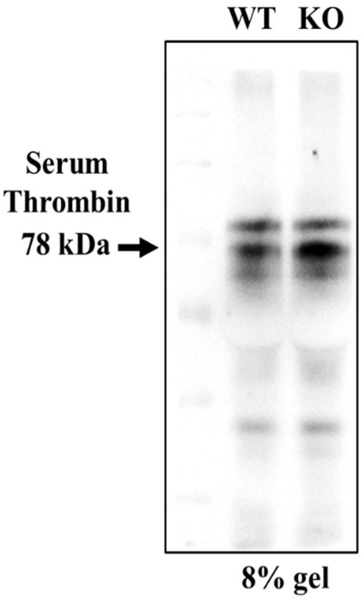

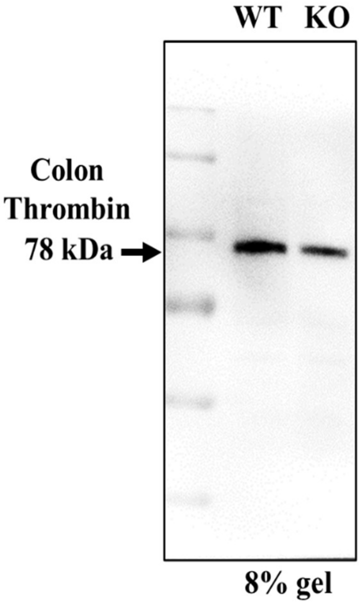

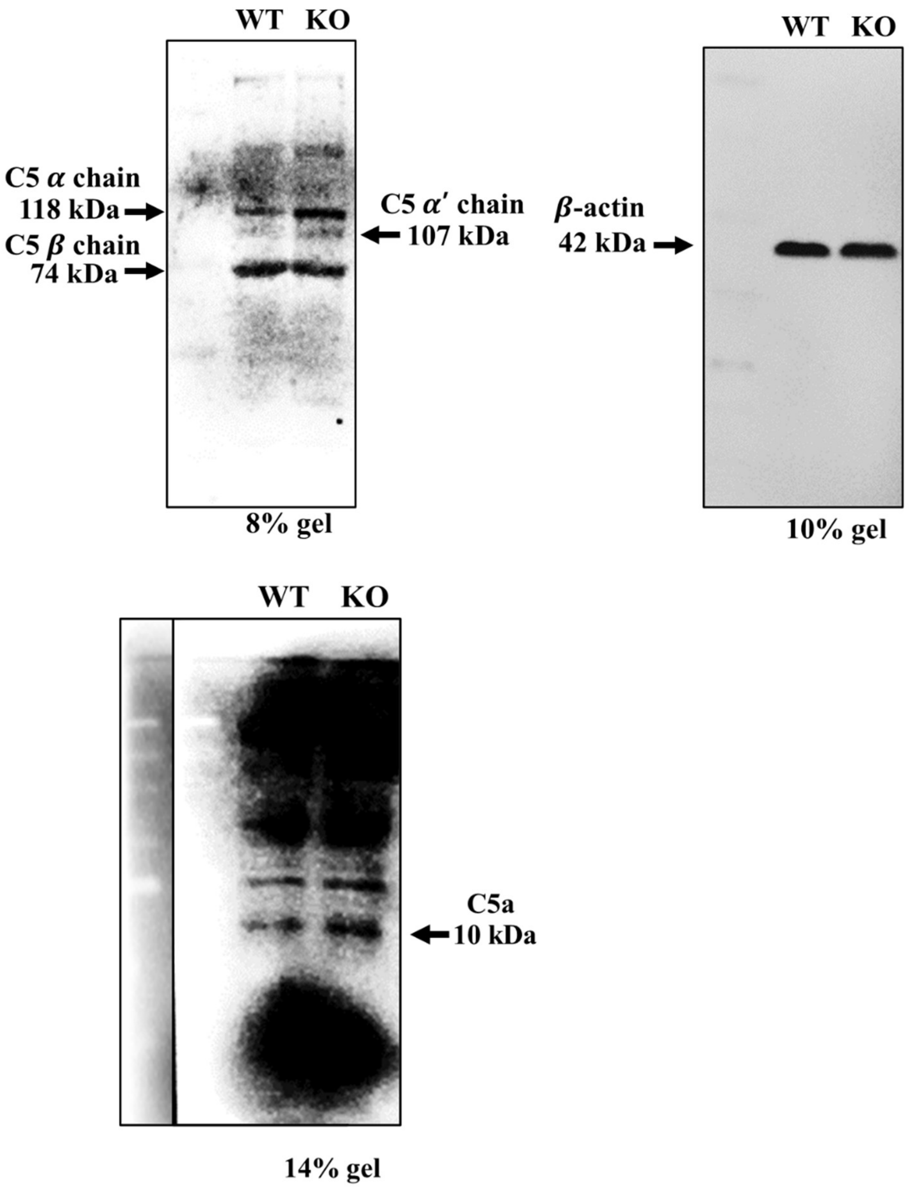

Original images of the gels in Figure 7A.

Supplement Fig. 12

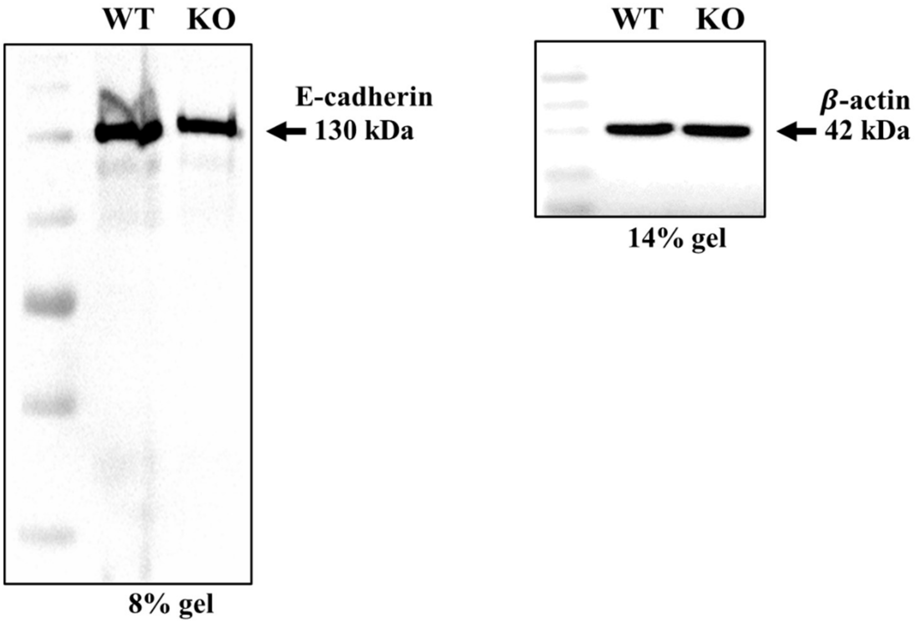

Original images of the gels in Figure 8A.
